# Estimation for Entropy and Parameters of Generalized Bilal Distribution under Adaptive Type II Progressive Hybrid Censoring Scheme

**DOI:** 10.3390/e23020206

**Published:** 2021-02-08

**Authors:** Xiaolin Shi, Yimin Shi, Kuang Zhou

**Affiliations:** 1 School of Electronics Engineering, Xi’an University of Posts and Telecommunications, Xi’an 710121, China; linda20016@163.com; 2 School of Mathematics and Statistics, Northwestern Polytechnical University, Xi’an 710072, China; kzhoumath@nwpu.edu.cn

**Keywords:** entropy, generalized Bilal distribution, adaptive Type-II progressive hybrid censoring scheme, maximum likelihood estimation, Bayesian estimation, Lindley’s approximation, confidence interval, Markov chain Monte Carlo method

## Abstract

Entropy measures the uncertainty associated with a random variable. It has important applications in cybernetics, probability theory, astrophysics, life sciences and other fields. Recently, many authors focused on the estimation of entropy with different life distributions. However, the estimation of entropy for the generalized Bilal (GB) distribution has not yet been involved. In this paper, we consider the estimation of the entropy and the parameters with GB distribution based on adaptive Type-II progressive hybrid censored data. Maximum likelihood estimation of the entropy and the parameters are obtained using the Newton–Raphson iteration method. Bayesian estimations under different loss functions are provided with the help of Lindley’s approximation. The approximate confidence interval and the Bayesian credible interval of the parameters and entropy are obtained by using the delta and Markov chain Monte Carlo (MCMC) methods, respectively. Monte Carlo simulation studies are carried out to observe the performances of the different point and interval estimations. Finally, a real data set has been analyzed for illustrative purposes.

## 1. Introduction

To analyze and evaluate the reliability of products, life tests are often carried out. For products with long lives and high reliability, a censoring scheme is often adopted during the test to save on time and costs. Two commonly used censoring schemes are Type-I and Type-II censoring, but these two censoring schemes do not have the flexibility of allowing the removal of units at points other than the terminal point of the experiment. To allow for more flexibility in removing surviving units from the test, more general censoring approaches are required. The progressive Type-II censoring scheme is appealing and has attracted much attention in the literature. This topic can be found in [1]. One may also refer to [2] for a comprehensive review on progressive censoring. One drawback of the Type-II progressive censoring scheme is that the length of the experiment may be quite long for long-life products. Therefore, Kundu and Joarder [3] proposed a Type-II progressive hybrid censoring scheme where the experiment terminates at a pre-specified time. However, for the Type-II progressive hybrid censoring scheme, the drawback is that the effective sample size is a random variable, which may be very small or even zero. To strike a balance between the total testing time and the efficiency in statistical inference, Ng et al. [4] introduced an adaptive Type-II progressive hybrid censoring scheme (ATII-PHCS). This censoring scheme is described as follows. Suppose that n units are placed on test and $X_{1},X_{2},\ldots ,X_{n}$ denote the corresponding lifetimes from a distribution with the cumulative distribution function (CDF) F(x) and the probability density function (PDF) f(x). The number of observed failures *m* and time *T* are specified in advance and m<n. At the first failure time $X_{1:m:n}$, $R_{1}$ units are randomly removed from the remaining n−1 units. Similarly, at the second failure time $X_{2:m:n}$, $R_{2}$ units from the remaining $n-2-R_{1}$ units are randomly removed, and so on. If the *m*th failure occurs before time T (i.e., $X_{m:m;n}<T$), the test terminates at time $X_{m:m:n}$ and all remaining $R_{m}$ units are removed, where $R_{m}=n-m-\sum_{i=1}^{m-1}R_{i}$ and $R_{i}$ is specified in advance (i=1,2,…,m). If the *J*th failure occurs before time T (i.e., $X_{J:m:n}<T<X_{J+1:m:n}$ where J+1<m), then we will not withdraw any units from the test by setting $R_{J+1}=R_{J+2}=\ldots =R_{m-1}=0$, and the test will continue until the failure unit number reaches the prefixed number *m*. At the time of the *m*th failure, all remaining $R_{m}$ units are removed and the test terminates, where $R_{m}=n-m-\sum_{i=1}^{J}R_{i}$.

The main advantage of ATII-PHCS is that it speeds up the test when the test duration exceeds the predetermined time T and ensures we get the effective number of failures *m*. It also illustrates how an experimenter can control the experiment. If one is interested in getting observations early, one will remove fewer units (or even none). For convenience, we let $X_{i}=X_{i:m:n},i=1,2,\ldots ,m$. After the above test, we get one of the following observation data cases:

Case I: $\left(X_{1},R_{1}^{}),(X_{2},R_{2}^{}),\ldots ,(X_{m},R_{m}^{}\right)$ if $X_{m}<T$, where $R_{m}=n-\sum_{i=1}^{m-1}R_{i}-m$.
Case II: $\left(X_{1},R_{1}^{}),(X_{2},R_{2}^{}),\ldots ,(X_{J},R_{J}^{}),(X_{J+1},0),\ldots ,(X_{m-1},0),(X_{m},R_{m}\right)$ if $X_{J}<T<X_{J+1}$ and J<m, where $R_{m}=n-m-\sum_{i=1}^{J}R_{i}$.

The ATII-PHCS has been studied in recent years. Mazen et al. [5] discussed the statistical analysis of the Weibull distribution under an adaptive Type-II progressive hybrid censoring scheme. Zhang et al. [6] investigated the maximum likelihood estimations (MLEs) of the unknown parameters and acceleration factors in the step-stress accelerated life test, based on the tampered failure rate model with ATII-PHC samples. Cui et al. [7] studied the point and interval estimates of the parameters from the Weibull distribution, based on adaptive Type-II progressive hybrid censored data in a constant-stress accelerated life test. Ismail [8] proposed that the MLE of the Weibull distribution parameters and the acceleration factor were derived based on ATII-PHC schemes under a step-stress partially accelerated life test model. The statistical inference of the dependent competitive failure system under the constant-stress accelerated life test with ATII-PHC data was studied by Zhang et al. [9]. Under an adaptive Type-II progressive censoring scheme, Ye et al. [10] investigated the general statistical properties and then used the maximum likelihood technique to estimate the parameters of the extreme value distribution. Some other studies on the statistical inference of life models using ATII-PHCS were presented by Sobhi and Soliman [11] and Nassar et al. [12]. Xu and Gui [13] studied entropy estimation for the two-parameter inverse Weibull distribution under adaptive type-II progressive hybrid censoring schemes.

Entropy measures the uncertainty associated with a random variable. Let X be a random variable having a continuous CDF F(x) and PDF f(x). Then, the Shannon entropy is defined as

(1) $$H(f)=-\int_{-\infty }^{+\infty }f(x)\ln f(x)dx.$$

In recent years, several scholars have studied the entropy estimation of different life distributions. Kang et al. [14] investigated the entropy estimators of a double exponential distribution based on multiply Type-II censored samples. Cho et al. [15] derived an estimation for the entropy function of a Rayleigh distribution based on doubly generalized Type-II hybrid censored samples. Baratpour et al. [16] developed the entropy of the upper record values and provided several upper and lower bounds for this entropy by using the hazard rate function. Cramer and Bagh [17] discussed the entropy of the Weibull distribution under progressive censoring. Cho et al. [18] obtained estimators for the entropy function of the Weibull distribution based on a generalized Type-II hybrid censored sample. Yu et al. [19] studied statistical inference in the Shannon entropy of the inverse Weibull distribution under progressive first-failure censoring.

In addition to the above-mentioned life distributions, the generalized Bilal (GB) distribution is also an important life distribution for analyzing lifetime data. The PDF and the CDF of the GB distribution, respectively, are given as

(2) $$f(x;\beta ,\lambda )=6\beta \lambda x_{}^{\lambda -1}\exp(-2\beta x_{}^{\lambda })[1-\exp(-\beta x_{}^{\lambda })],x>0,\beta >0,\lambda >0,$$

(3) $$F(x;\beta ,\lambda )=1-\exp(-2\beta x_{}^{\lambda })[3-2\exp(-\beta x_{}^{\lambda })],x>0,\beta >0,\lambda >0,$$

The Shannon entropy of the GB distribution is given by

$$H(f)=H(\beta ,\lambda )=2.5+\gamma -\ln(27/4)-\ln(\lambda \beta^{\frac{1}{\lambda }})+\frac{1}{\lambda }(\ln(9/8)-\gamma ),\beta >0,\lambda >0,$$

where γ denotes the Euler–Mascheroni constant and γ=0.5772.

The GB distribution was first introduced by Abd-Elrahman [20]. He investigated the properties of the probability density and failure rate function of this distribution. A comprehensive mathematical treatment of the GB distribution was provided, and the maximum likelihood estimations of unknown parameters were derived under the complete sample. Abd-Elrahman [21] provided the MLEs and Bayesian estimations of the unknown parameters and the reliability function based on a Type-II censored sample. Since the failure rate function of GB distribution has an upside-down bathtub shape, and it can also be monotonically decreasing or monotonically increasing at some selected values of the shape parameters λ, the GB model is very useful in survival analysis and reliability studies.

To the best of our knowledge, there has been no published work on the estimation of the entropy and parameters of GB distribution under an ATII-PHCS. As such, these issues are considered in this paper. The main objective of this paper is to provide the estimation of the entropy and unknown parameters of GB distribution under an ATII-PHCS by using the frequency and Bayesian methods.

The rest of this paper is organized as follows. In Section 2, the MLEs of the parameters and entropy of GB distribution are obtained, and approximate confidence intervals are constructed using the ATII-PHC data. In Section 3, the Bayesian estimation of the parameters and entropy under three different loss functions are provided using Lindley’s approximation method. In addition, the Bayesian credible intervals of the parameters and entropy are also obtained by using the Markov chain Monte Carlo (MCMC) method. In Section 4, Monte Carlo simulations are carried out to investigate the performance of different point estimates and interval estimates. In Section 5, a real data set is analyzed for illustrative purposes. Some conclusions are presented in Section 6.

## 2. Maximum Likelihood Estimation

In this section, the MLE and approximate confidence intervals of the parameters and entropy of GB distribution will be discussed under the ATII-PHCS. Based on the data in Case I and Case II, the likelihood functions can be respectively written as

(4) $$Case\;I:L_{I}(\beta ,\lambda |\vec{x})\propto \prod_{i=1}^{m}f(x_{i};\beta ,\lambda )[1-F(x_{i};\beta ,\lambda )){]}^{R_{i}^{}},$$

(5) $$Case\;II:L_{II}(\beta ,\lambda |\vec{x})\propto \prod_{i=1}^{m}f(x_{i};\beta ,\lambda ))\prod_{i=1}^{J}{\left[1-F(x_{i};\beta ,\lambda )\right]}^{R_{i}^{}}{\left[1-F(x_{m};\beta ,\lambda )\right]}^{n-m-\sum_{i=1}^{J}R_{i}},$$

where $\vec{x}=(x_{1},x_{2},\ldots ,x_{m})$.

By combining $L_{I}(\beta ,\lambda |\vec{x})\;\mathrm{and}\;L_{II}(\beta ,\lambda |\vec{x})$, the likelihood functions can be written uniformly as

(6) $$\begin{array}{l} L(\beta ,\lambda |\vec{x})\propto \prod_{i=1}^{m}f(x_{i};\beta ,\lambda ))\prod_{i=1}^{D}{\left[1-F(x_{i};\beta ,\lambda )\right]}^{R_{i}^{}}{\left[1-F(x_{m};\beta ,\lambda )\right]}^{R^{*}}=\\ =\prod_{i=1}^{m}6\beta \lambda x_{i}^{\lambda -1}\exp(-2\beta x_{i}^{\lambda })[1-\exp(-\beta x_{i}^{\lambda })]\prod_{i=1}^{D}\left[\exp(-2\beta x_{i}^{\lambda }\right)(3-2\exp(-\beta x_{i}^{\lambda })){]}^{R_{i}^{}}\times {\left[\exp(-2\beta x_{m}^{\lambda })(3-2\exp(-\beta x_{m}^{\lambda }))\right]}^{R^{*}}, \end{array}$$

where $R_{}^{*}=n-m-\sum_{i=1}^{D}R_{i}$ and, for Case I, $D=m,R^{*}=0$, and for Case II, $D=J,R^{*}=n-m-\sum_{i=1}^{J}R_{i}$.

The log-likelihood function is given by

(7) $$\begin{array}{l} l=\ln L(\beta ,\lambda |\vec{x})\propto m\ln(6\beta \lambda )+\sum_{i=1}^{m}[(\lambda -1)\ln x_{i}-2\beta x_{i}^{\lambda }+\ln(1-\exp(-\beta x_{i}^{\lambda }))]\;+\\ +\sum_{i=1}^{D}[-2R_{i}\beta x_{i}^{\lambda }+R_{i}\ln(3-2\exp(-\beta x_{i}^{\lambda }))]-2R_{}^{*}\beta x_{m}^{\lambda }+R_{}^{*}\ln(3-2\exp(-\beta x_{m}^{\lambda })). \end{array}$$

By taking the first partial derivative of the log-likelihood function with regard to β and λ and equating them to zero, the following results can be obtained:

(8) $$\frac{\partial l}{\partial \beta }=\frac{m}{\beta }+\sum_{i=1}^{m}\left[-3x_{i}^{\lambda }+x_{i}^{\lambda }{\left[y_{1}(\theta )\right]}^{-1}\right]+\sum_{i=1}^{D}\left[-3R_{i}x_{i}^{\lambda }+3R_{i}x_{i}^{\lambda }{\left[y_{2}(\theta )\right]}^{-1}\right]-3R_{}^{*}x_{m}^{\lambda }+3R_{}^{*}x_{m}^{\lambda }{\left[y_{3}(\theta )\right]}^{-1}=0,$$

(9) $$\begin{array}{l} \frac{\partial l}{\partial \lambda }=\frac{m}{\lambda }+\sum_{i=1}^{m}\left[\ln x_{i}-3\beta x_{i}^{\lambda }\ln x_{i}+\beta x_{i}^{\lambda }\ln x_{i}{\left[y_{1}(\theta )\right]}^{-1}\right]+\sum_{i=1}^{D}\left[-3R_{i}\beta x_{i}^{\lambda }\ln x_{i}+3R_{i}\beta x_{i}^{\lambda }\ln x_{i}{\left[y_{2}(\theta )\right]}^{-1}\right]-\\ -3R_{}^{*}\beta x_{m}^{\lambda }\ln x_{m}+3R_{}^{*}\beta x_{m}^{\lambda }\ln x_{m}{\left[y_{3}(\theta )\right]}^{-1}=0, \end{array}$$

where $\theta =(\beta ,\lambda ),\;y_{1}(\theta )=1-\exp(-\beta x_{i}^{\lambda }),y_{2}(\theta )=3-2\exp(-\beta x_{i}^{\lambda }),y_{3}(\theta )=3-2\exp(-\beta x_{m}^{\lambda }).$

The MLEs of β and λ can be obtained by solving Equations (7) and (8), but the above two equations do not yield an analytical solution. Thus, we use the Newton–Raphson iteration method to obtain the MLEs of the parameters. For this purpose, we firstly calculate the second partial derivatives of the log-likelihood function with regard to β and λ:

(10) $$\frac{\partial^{2}l}{\partial \beta^{2}}=-\frac{m}{\beta^{2}}-\sum_{i=1}^{m}[x_{i}^{2\lambda }\exp(-\beta x_{i}^{\lambda })]{\left[y_{1}(\theta )\right]}^{-2}-\sum_{i=1}^{D}6R_{i}x_{i}^{2\lambda }\exp(-\beta x_{i}^{\lambda }){\left[y_{2}(\theta )\right]}^{-2}-6R_{}^{*}x_{m}^{2\lambda }\exp(-\beta x_{m}^{\lambda }){\left[y_{3}(\theta )\right]}^{-2},$$

(11) $$\begin{array}{l} \frac{\partial^{2}l}{\partial \beta \partial \lambda }=\sum_{i=1}^{m}\left[-3x_{i}^{\lambda }\ln x_{i}+x_{i}^{\lambda }\ln x_{i}(y_{1}{\left(\theta \right)}^{-1}[1-\beta x_{i}^{\lambda }\exp(-\beta x_{i}^{\lambda }\right){\left(y_{1}(\theta )\right)}^{-1}]+\\ +\sum_{i=1}^{D}\left[-3R_{i}x_{i}^{\lambda }\ln x_{i}+3R_{i}x_{i}^{\lambda }\ln x_{i}(y_{2}{\left(\theta \right)}^{-1}(1-2\beta x_{i}^{\lambda }\exp(-\beta x_{i}^{\lambda }\right){\left(y_{2}(\theta )\right)}^{-1})]-\\ -3R_{}^{*}x_{m}^{\lambda }+3R_{}^{*}x_{m}^{\lambda }\ln x_{m}{\left[y_{3}(\theta )\right]}^{-1}[1-2\beta x_{m}^{\lambda }\exp(-\beta x_{m}^{\lambda }){\left(y_{3}(\theta )\right)}^{-1})], \end{array}$$

(12) $$\begin{array}{l} \frac{\partial^{2}l}{\partial \lambda^{2}}=-\frac{m}{\lambda^{2}}+\sum_{i=1}^{m}\left[\beta x_{i}^{\lambda }{\left(\ln x_{i}\right)}^{2}[-3+{\left(y_{1}(\theta )\right)}^{-1}]-\beta^{2}x_{i}^{2\lambda }{\left(\ln x_{i}\right)}^{2}\exp(-\beta x_{i}^{\lambda }){\left(y_{1}(\theta )\right)}^{-2}\right]\\ +\sum_{i=1}^{D}\left[-3R_{i}\beta x_{i}^{\lambda }{\left(\ln x_{i}\right)}^{2}(1-{\left(y_{2}(\theta )\right)}^{-1})-6R_{i}\beta^{2}x_{i}^{2\lambda }{\left(\ln x_{i}\right)}^{2}\exp(-\beta x_{i}^{\lambda }){\left(y_{2}(\theta )\right)}^{-2}\right]\\ -3R_{}^{*}\beta x_{m}^{\lambda }{\left(\ln x_{m}\right)}^{2}(1-{\left(y_{3}(\theta )\right)}^{-1})-6R_{}^{*}\beta^{2}x_{m}^{2\lambda }{\left(\ln x_{m}\right)}^{2}\exp(-\beta x_{m}^{\lambda }){\left(y_{3}(\theta )\right)}^{-2}. \end{array}$$

Let $I(\beta ,\lambda )=\left[\begin{array}{cc} I_{11} & I_{12}\\ I_{21} & I_{22} \end{array}\right]$, where

(13) $$I_{11}=-\frac{\partial^{2}l}{\partial \beta^{2}},\;I_{22}=-\frac{\partial^{2}l}{\partial \lambda^{2}},I_{12}=I_{21}=-\frac{\partial^{2}l}{\partial \beta \partial \lambda }.\;$$

On the basis of the above calculation results, we can implement the Newton–Raphson iteration method to obtain the MLEs of unknown parameters. The specific steps of this iteration method can be seen in Appendix B. After obtaining the MLE $\hat{\beta }$ and $\hat{\lambda }$ of the parameters β and λ, using the invariant property of MLEs, the MLE of the entropy H (f) for the generalized Bilal distribution is given by

(14) $$\hat{H}(f)=2.5+\gamma -\ln(27/4)-\frac{1}{\hat{\lambda }}\ln\hat{\beta }-\ln\hat{\lambda }+\frac{1}{\hat{\lambda }}(\ln(9/8)-\gamma ).$$

### Approximate Confidence Interval

In this subsection, the approximate confidence intervals of the parameters β,λ and the Shannon entropy H (f) are derived. Based on regularity conditions, the MLEs $\left(\hat{\beta },\hat{\lambda }\right)$ are an approximately bivariate normal distribution $N((\beta ,\lambda ),\;I^{-1}(\hat{\beta },\hat{\lambda }))$, where the covariance matrix $I^{-1}(\beta ,\lambda )$ is an estimation of $I^{-1}(\beta ,\lambda )$ and $\;I^{-1}(\hat{\beta },\hat{\lambda })={\left[\begin{array}{cc} I_{11} & I_{12}\\ I_{21} & I_{22} \end{array}\right]}_{\left(\beta ,\lambda )=(\hat{\beta },\hat{\lambda }\right)}^{-1}$, $I_{11},I_{22},I_{12}$ and $I_{21}$ are given by Equations (10)–(13), respectively.

Thus, the approximate 100(1−α)% two-sided confidence intervals (CIs) for parameters β,λ are given by

(15) $$\left(\hat{\beta }\pm z_{\alpha /2}\sqrt{Var(\hat{\beta })}\right),\left(\hat{\lambda }\pm z_{\alpha /2}\sqrt{Var(\hat{\lambda })}\right),$$

where $z_{\alpha /2}$ is the upper α/2 percentile of the standard normal distribution and $Var(\hat{\beta })$, $Var(\hat{\lambda })$ are the main diagonal elements of the matrix $\;I^{-1}(\hat{\beta },\hat{\lambda })$.

Next, we use the delta method to obtain the asymptotic confidence interval of the entropy H (f). The delta method is a general approach to compute CIs for functions of MLEs. Under a progressive Type-II censored sample, the authors of [22] used the delta method to study the estimation of a new Weibull–Pareto distribution. The authors of [23] also used this method to investigate the estimation of the two-parameter bathtub lifetime model.

Let $M^{T}=(\frac{\partial H(f)}{\partial \beta },\frac{\partial H(f)}{\partial \lambda })$, where $\frac{\partial H(f)}{\partial \beta }=-\frac{1}{\beta \lambda },\;\frac{\partial H(f)}{\partial \lambda }=\frac{1}{\lambda^{2}}\ln\beta -\frac{1}{\lambda }-\frac{1}{\lambda^{2}}(\ln\frac{9}{8}-\gamma )$.

Then, the approximate estimates of $var(\hat{H}(f))$ is given by

$$v\hat{a}r(\hat{H}(f))=[M^{T}\;I^{-1}(\beta ,\lambda )M]|{}_{\left(\beta ,\lambda )=(\hat{\beta },\hat{\lambda }\right)},\;$$

where $\hat{\beta }$ and $\hat{\lambda }$ are the MLEs of β and λ, respectively, and $I^{-1}(\beta ,\lambda )$ denotes the inverse of the matrix $I(\beta ,\lambda )=\left[\begin{array}{cc} I_{11} & I_{12}\\ I_{21} & I_{22} \end{array}\right]$. The elements of the matrix I(β,λ) are given by Equations (10)–(13), respectively. Thus, $\frac{\hat{H}(f)-H(f)}{\sqrt{v\hat{a}r(\hat{H}(f))}}$ is asymptotically distributed as N(0,1). The asymptotic 100(1−α)% CI for the entropy H (f) is given by

$$\left(\hat{H}(f)\pm Z_{\alpha /2}\sqrt{v\hat{a}r(\hat{H}(f))}\right)$$

where $z_{\alpha /2}$ is the upper α/2 percentile of the standard normal distribution.

## 3. Bayesian Estimation

In this section, we discuss the Bayesian point estimation of the parameters and entropy H (f) for generalized Bilal distribution using Lindley’s approximation method under symmetric as well as asymmetric loss functions. Furthermore, the Bayesian CI of the parameters and entropy are also derived by using the Markov chain Monte Carlo method.

### 3.1. Loss Functions and Posterior Distribution

Choosing the loss function is an important part in the Bayesian inference. The commonly used symmetric loss function is the squared error loss (SEL) function, which is defined as

(16) $$L_{1}(U,\hat{U})=(\hat{U}-U{)}^{2}.$$

Two popular asymmetric loss functions are the Linex loss (LL) and general entropy loss (EL) functions, which are respectively given by

(17) $$L_{2}(U,\hat{U})=\exp(h(\hat{U}-U))-h\;(\hat{U}-U)-1,\;h\neq 0,$$

(18) $$L_{3}(U,\hat{U})\propto {\left(\frac{\hat{U}}{U}\right)}^{q}-q\ln\left(\frac{\hat{U}}{U}\right)-1,\;q\neq 0.$$

Here, U=U(β,λ) is any function of β and λ, and $\hat{U}$ is an estimate of U. The constant h and q represent the weight of errors on different decisions. Under the above loss functions, the Bayesian estimate of function U can be calculated by

(19) $${\hat{U}}_{S}=E(U|\vec{x}).$$

(20) $${\hat{U}}_{L}=-\frac{1}{h}\ln[E(\exp(-hU)|\vec{x})],\begin{array}{cc} & h\neq 0 \end{array}.$$

(21) $${\hat{U}}_{E}={\left[E(U^{-q}|\vec{x})\right]}^{-1/q},\begin{array}{cc} & q\neq 0 \end{array}.$$

To derive the Bayesian estimates of the function U(β,λ), we consider prior distributions of the unknown parameters β and λ as independent Gamma distributions Ga (a,b)  and Ga (c,d), respectively. Therefore, the joint prior distribution of β and λ becomes

$$\pi (\beta ,\lambda )=\frac{b^{a}\beta^{a-1}}{\Gamma (a)}\exp(-b\beta )\;\frac{d^{\;c}\lambda^{c-1}}{\Gamma (c)}\exp(-d\lambda ),\;(\beta ,\lambda ,a,b,c,d>0).$$

Based on the likelihood function $L(\beta ,\lambda |\vec{x})$ and the joint prior distribution of β and λ, the joint posterior density of parameters β and λ can be written as

(22) $$\begin{array}{ll} \pi (\beta ,\lambda |\vec{x})= & \frac{\pi (\beta ,\lambda )L(\beta ,\lambda |\vec{x})}{\int_{0}^{\infty }\int_{0}^{\infty }\pi (\beta ,\lambda )L(\beta ,\lambda |\vec{x})d\beta d\lambda }\\ & \propto \pi (\beta ,\lambda )L(\beta ,\lambda |\vec{x})\\ & =\beta^{a-1}\exp(-b\beta )\lambda^{c-1}\exp(-d\lambda )A_{1}(\beta ,\lambda )A_{2}(\beta ,\lambda )A_{3}(\beta ,\lambda ), \end{array}$$

where

$$A_{1}(\beta ,\lambda )=\prod_{i=1}^{m}6\beta \lambda x_{i}^{\lambda -1}\exp(-2\beta x_{i}^{\lambda })[1-\exp(-\beta x_{i}^{\lambda })],$$

$$A_{2}(\beta ,\lambda )=\prod_{i=1}^{D}\left[\exp(-2\beta x_{i}^{\lambda }\right)(3-2\exp(-\beta x_{i}^{\lambda })){]}^{R_{i}^{}},\;$$

$$A_{3}(\beta ,\lambda )={\left[\exp(-2\beta x_{m}^{\lambda })(3-2\exp(-\beta x_{m}^{\lambda }))\right]}^{R^{*}}.$$

Therefore, the Bayesian estimate of U(β,λ) under the SEL, LL and GEL functions are respectively given by

(23) $${\hat{U}}_{S}(\beta ,\lambda )=\frac{\int_{0}^{\infty }\int_{0}^{\infty }U(\beta ,\lambda )\pi (\beta ,\lambda )L(\beta ,\lambda |\vec{x})d\beta d\lambda }{\int_{0}^{\infty }\int_{0}^{\infty }\pi (\beta ,\lambda )L(\beta ,\lambda |\vec{x})d\beta d\lambda },$$

(24) $${\hat{U}}_{L}(\beta ,\lambda )=-\frac{1}{h}\ln\left[\frac{\int_{0}^{\infty }\int_{0}^{\infty }\exp(-hU(\beta ,\lambda ))\pi (\beta ,\lambda )L(\beta ,\lambda |\vec{x})d\beta d\lambda }{\int_{0}^{\infty }\int_{0}^{\infty }\pi (\beta ,\lambda )L(\beta ,\lambda |\vec{x})d\beta d\lambda }\right],$$

(25) $${\hat{U}}_{E}(\beta ,\lambda )={\left[\frac{\int_{0}^{\infty }\int_{0}^{\infty }{\left(U(\beta ,\lambda )\right)}^{-q}\pi (\beta ,\lambda )L(\beta ,\lambda |\vec{x})d\beta d\lambda }{\int_{0}^{\infty }\int_{0}^{\infty }\pi (\beta ,\lambda )L(\beta ,\lambda |\vec{x})d\beta d\lambda }\right]}^{-\;\frac{1}{q}}.$$

### 3.2. Lindley’s Approximation

From Equations (23)–(25), it is observed that all of these estimates of the U(β,λ) are in the form of the ratio of two integrals which cannot be reduced to a closed form. Therefore, we use Lindley’s approximation method to obtain the Bayesian estimates. If we let $\theta =(\theta_{1},\theta_{2})$, then the posterior expectation of a function $U\;(\theta_{1},\theta_{2})$ can be approximated as in [18]:

(26) $$\hat{U}=U({\hat{\theta }}_{1},{\hat{\theta }}_{2})+0.5(A+z_{30}B_{12}+z_{03}B_{21}+z_{21}C_{12}+z_{12}C_{21})+p_{1}A_{12}+p_{2}A_{21},$$

where $U({\hat{\theta }}_{1},{\hat{\theta }}_{2})$ is the MLE of $U(\theta_{1},\theta_{2})$ and

$$A=\sum_{i=1}^{2}\sum_{j=1}^{2}u_{ij}\tau_{ij},B_{ij}=(u_{i}\tau_{ii}+u_{j}\tau_{ij})\tau_{ii},C_{ij}=3u_{i}\tau_{ii}\tau_{ij}+u_{j}(\tau_{ii}\tau_{jj}+2\tau_{ij}^{2}),$$

$$\begin{array}{l} p_{i}=\frac{\partial p}{\partial \theta_{i}},u_{i}=\frac{\partial U}{\partial \theta_{i}},u_{ij}=\frac{\partial^{2}U}{\partial \theta_{i}\partial \theta_{j}},p=\ln\pi (\theta_{1},\theta_{2}),A_{ij}=u_{i}\tau_{ii}+u_{j}\tau_{ji},\\ z_{ij}=\frac{\partial^{i+j}l(\theta_{1},\theta_{2})}{\partial \theta_{1}{}^{i}\partial \theta_{2}{}^{j}},\;i,j=0,1,2,3,i+j=3, \end{array}$$

where l denotes the log-likelihood function and $\tau_{ij}(i,j)$ denotes the (i,j)-th element of the matrix ${\left[-\partial^{2}l/\partial \theta_{1}{}^{i}\partial \theta_{2}{}^{j}\right]}^{-1}$. All terms are estimated by MLEs of the parameters $\theta_{1}$ and $\theta_{2}$.

Based on the above equations, we have

(27) $$\begin{array}{l} z_{30}=\frac{\partial^{3}l}{\partial \beta^{3}}=\frac{2m}{\beta^{3}}+\sum_{i=1}^{m}\{x_{i}^{3\lambda }\exp(-\beta x_{i}^{\lambda }){\left(y_{1}(\theta )\right)}^{-2}[1+2{\left(y_{1}(\theta )\right)}^{-1}\exp(-\beta x_{i}^{\lambda })]\}\\ +\sum_{i=1}^{D}\left\{6R_{i}x_{i}^{3\lambda }\exp(-\beta x_{i}^{\lambda }\right){\left(y_{2}(\theta )\right)}^{-2}[1+4\exp(-\beta x_{i}^{\lambda }){\left(y_{2}(\theta )\right)}^{-1}]\}\\ +6R_{}^{*}x_{m}^{3\lambda }\exp(-\beta x_{m}^{\lambda }){\left(y_{3}(\theta )\right)}^{-2}[1+4\exp(-\beta x_{m}^{\lambda }){\left(y_{3}(\theta )\right)}^{-1}]. \end{array}$$

(28) $$\begin{array}{l} z_{03}=\frac{\partial^{3}l}{\partial \lambda^{3}}=\frac{2m}{\lambda^{3}}+\sum_{i=1}^{m}\{\beta x_{i}^{\lambda }{\left(\ln x_{i}\right)}^{3}(-3+{\left(y_{1}(\theta )\right)}^{-1})-\beta^{2}x_{i}^{2\lambda }{\left(\ln x_{i}\right)}^{3}\exp(-\beta x_{i}^{\lambda })\;{\left(y_{1}(\theta )\right)}^{-2}\\ \times \;[3-\beta x_{i}^{\lambda }-2\beta x_{i}^{\lambda }\exp(-\beta x_{i}^{\lambda }){\left(y_{1}(\theta )\right)}^{-1}]\}+\sum_{i=1}^{D}\{[-3R_{i}\beta x_{i}^{\lambda }{\left(\ln x_{i}\right)}^{3}[1-{\left(y_{2}(\theta )\right)}^{-1}]+\\ +6R_{i}\beta^{2}x_{i}^{2\lambda }{\left(\ln x_{i}\right)}^{3}\exp(-\beta x_{i}^{\lambda }){\left(y_{2}(\theta )\right)}^{-2}(-3+\beta x_{i}^{\lambda }+4\beta x_{i}^{\lambda }{\left(y_{2}(\theta )\right)}^{-1}\exp(-\beta x_{i}^{\lambda }))\}+\\ +3R_{}^{*}\beta x_{m}^{\lambda }{\left(\ln x_{m}\right)}^{3}[-1+{\left(y_{3}(\theta )\right)}^{-1}]+6R_{}^{*}\beta^{2}x_{m}^{2\lambda }{\left(\ln x_{m}\right)}^{3}\exp(-\beta x_{m}^{\lambda }){\left(y_{3}(\theta )\right)}^{-2}[-3+\beta x_{m}^{\lambda }+4\beta x_{m}^{\lambda }{\left(y_{3}(\theta )\right)}^{-1}\exp(-\beta x_{m}^{\lambda })]. \end{array}$$

(29) $$\begin{array}{l} z_{21}=\frac{\partial^{3}l}{\partial \beta^{2}\partial \lambda }=\sum_{i=1}^{m}\left[-x_{i}^{2\lambda }\ln x_{i}\exp(-\beta x_{i}^{\lambda }){\left(y_{1}(\theta )\right)}^{-2})[2-\beta x_{i}^{\lambda }-2\beta x_{i}^{\lambda }\exp(-\beta x_{i}^{\lambda }){\left(y_{1}(\theta )\right)}^{-1}\right]\\ -\sum_{i=1}^{D}\left[6R_{i}x_{i}^{2\lambda }\ln x_{i}\exp(-\beta x_{i}^{\lambda }\right){\left(y_{2}(\theta )\right)}^{-2}[2-\beta x_{i}^{\lambda }-4\beta x_{i}^{\lambda }\exp(-\beta x_{i}^{\lambda }){\left(y_{2}(\theta )\right)}^{-1}]\\ -6R_{}^{*}x_{m}^{2\lambda }\ln x_{m}\exp(-\beta x_{m}^{\lambda }){\left(y_{3}(\theta )\right)}^{-2}][2-\beta x_{m}^{\lambda }-4\beta x_{m}^{\lambda }{\left(y_{3}(\theta )\right)}^{-1}\exp(-\beta x_{m}^{\lambda })]. \end{array}$$

(30) $$\begin{array}{l} z_{12}=\frac{\partial^{3}l}{\partial \beta \partial \lambda^{2}}=\sum_{i=1}^{m}[-3x_{i}^{\lambda }{\left(\ln x_{i}\right)}^{2}+x_{i}^{\lambda }{\left(\ln x_{i}\right)}^{2}{\left(y_{1}(\theta )\right)}^{-1}\\ +\beta x_{i}^{2\lambda }{\left(\ln x_{i}\right)}^{2}\exp(-\beta x_{i}^{\lambda }){\left(y_{1}(\theta )\right)}^{-2}{\left[-3+\beta x_{i}^{\lambda }+y_{1}(\theta )\right)}^{-1}\beta x_{i}^{\lambda }\exp(-\beta x_{i}^{\lambda })]]+\sum_{i=1}^{D}\{-3R_{i}x_{i}^{\lambda }{\left(\ln x_{i}\right)}^{2}+3R_{i}x_{i}^{\lambda }{\left(\ln x_{i}\right)}^{2}{\left(y_{2}(\theta )\right)}^{-1}\\ +6\beta R_{i}x_{i}^{2\lambda }{\left(\ln x_{i}\right)}^{2}\exp(-\beta x_{i}^{\lambda }){\left(y_{2}(\theta )\right)}^{-2}[-3+\beta x_{i}^{\lambda }\exp(-\beta x_{i}^{\lambda })+4{\left(y_{2}(\theta )\right)}^{-1}\beta x_{i}^{\lambda }\exp(-\beta x_{i}^{\lambda })]\\ -3R_{}^{*}x_{m}^{\lambda }{\left(\ln x_{m}\right)}^{2}+3R_{}^{*}x_{m}^{\lambda }{\left(\ln x_{m}\right)}^{2}{\left(y_{3}(\theta )\right)}^{-1}\}\\ +6\beta R_{}^{*}x_{m}^{2\lambda }{\left(\ln x_{m}\right)}^{2}\exp(-\beta x_{m}^{\lambda }){\left(y_{3}(\theta )\right)}^{-2}[-3+\beta x_{m}^{\lambda }\exp(-\beta x_{m}^{\lambda })+4{\left(y_{3}(\theta )\right)}^{-1}\beta x_{m}^{\lambda }\exp(-\beta x_{m}^{\lambda })]. \end{array}$$

$$p_{1}=\frac{a-1}{\beta }-b,p_{2}=\frac{c-1}{\lambda }-d,$$

$$\tau_{11}=-\;\frac{z_{02}}{z_{20}z_{02}-z_{11}^{2}},\tau_{22}=-\;\frac{z_{20}}{z_{20}z_{02}-z_{11}^{2}},\tau_{12}=\tau_{21}=\frac{z_{11}}{z_{20}z_{02}-z_{11}^{2}},$$

$$\;z_{20}=\frac{\partial^{2}l}{\partial \beta^{2}},z_{11}=\frac{\partial^{2}l}{\partial \beta \partial \lambda },z_{02}=\frac{\partial^{2}l}{\partial \lambda^{2}},$$

where $\;z_{20},z_{11},z_{02}$ are given by Equations (10)–(12), respectively.

Based on Lindley’s approximation, we can derive the Bayesian estimation of the two parameters, β and λ, and the entropy under different loss functions.

#### 3.2.1. Squared Error Loss Function

When U (β,λ)=β or λ, the Bayesian estimations of the parameters β and λ under the SEL function are given by, respectively,

$${\hat{\beta }}_{S}=\hat{\beta }+0.5[\tau_{11}^{2}z_{30}+\tau_{21}\tau_{22}z_{03}+3\tau_{11}\tau_{12}z_{21}+(\tau_{11}\tau_{22}+2\tau_{21}^{2})z_{12}]+\tau_{11}p_{1}+\tau_{12}p_{2},\;$$

$${\hat{\lambda }}_{S}=\hat{\lambda }+0.5[\tau_{11}\tau_{12}z_{30}+\tau_{22}^{2}z_{03}+3\tau_{22}\tau_{21}z_{12}+(\tau_{11}\tau_{22}+2\tau_{21}^{2})z_{21}]+\tau_{21}p_{1}+\tau_{22}p_{2},\;$$

where $\hat{\beta }$ and $\hat{\lambda }$ are the MLEs of the parameters β and λ, respectively.

Similarly, the Bayesian estimation of the entropy can be derived. We notice that

$$\begin{array}{l} U(\beta ,\lambda )=H(\beta ,\lambda )=2.5+\gamma -\ln(27/4)-\ln\lambda -\frac{1}{\lambda }\ln\beta +\frac{1}{\lambda }(\ln(9/8)-\gamma ),\\ u_{1}=-\frac{1}{\beta \lambda },u_{2}=-\frac{1}{\lambda }+\frac{1}{\lambda^{2}}(\ln\beta -\ln(9/8)+\gamma ),\\ u_{11}=\frac{1}{\beta^{2}\lambda },u_{22}=\frac{1}{\lambda^{2}}-\frac{2}{\lambda^{3}}(\ln\beta -\ln(9/8)+\gamma ),\;u_{12}=u_{21}=\frac{1}{\beta \lambda^{2}}. \end{array}$$

Thus, the Bayesian estimation of the entropy H (f) under the SEL function is given by

(31) H^S(f)= H ^(f)+0.5[u11τ11+2u12τ12+u22τ22+z30(u1τ11+u2τ12)τ11+z03(u2τ22+u1τ12)τ22+z21(3u1τ11τ12+u2(τ11τ22+2τ122))+z12(3u2τ22τ21+u1(τ11τ22+2τ212))]+p1(u1τ11+u2τ21)+p2(u2τ22+u1τ12),

where $\hat{H}(f)$ represents the maximum likelihood estimate of H (f).

#### 3.2.2. Linex Loss Function

Based on Lindley’s approximation, the Bayesian estimations of two parameters, β and λ, and the entropy under the LL function can, respectively, be given by

$$\begin{array}{l} {\hat{\beta }}_{L}=-\frac{1}{h}\ln\{\exp(-h\hat{\beta })+0{.5[u}_{11}\tau_{11}{+u}_{1}\tau_{{}_{11}}^{2}z_{30}{+u}_{1}\tau_{21}\tau_{22}z_{03}{+3u}_{1}\tau_{11}\tau_{12}z_{21}\\ +(\tau_{11}\tau_{22}{+2u}_{1}\tau_{21}^{2}{)u}_{1}z_{12}]+u_{1}\tau_{11}p_{1}+u_{1}\tau_{12}p_{2}\} \end{array}$$

$$\begin{array}{l} {\hat{\lambda }}_{L}=-\frac{1}{h}\ln\{\exp(-h\hat{\lambda })+0{.5[u}_{22}\tau_{22}{+u}_{2}\tau_{11}\tau_{12}z_{30}{+u}_{2}\tau_{22}^{2}z_{03}+(\tau_{11}\tau_{22}+2\tau_{12}^{2}{)u}_{2}z_{21}\\ +3u_{2}\tau_{22}\tau_{21}z_{21}]+u_{2}\tau_{12}p_{1}+u_{2}\tau_{22}p_{2}\} \end{array}$$

(32) $$\begin{array}{l} {\hat{H}}_{L}(f)=-\;\frac{1}{h}\ln\{\exp[-h\hat{H}(f)]+0{.5[u}_{11}\tau_{11}{+2u}_{12}\tau_{12}+u_{22}\tau_{22}{+z}_{30}{(u}_{1}\tau_{11}{+u}_{2}\tau_{12})\tau_{11}{+z}_{03}{(u}_{2}\tau_{22}{+u}_{1}\tau_{21})\tau_{22}\\ {+z}_{21}{(3u}_{1}\tau_{11}\tau_{12}{+u}_{2}(\tau_{11}\tau_{22}+2\tau_{12}^{2}{))+z}_{12}{(3u}_{2}\tau_{22}\tau_{21}{+u}_{1}(\tau_{11}\tau_{22}+2\tau_{21}^{2}))]\\ +p_{1}{(u}_{1}\tau_{11}+u_{2}\tau_{21})+p_{2}{(u}_{2}\tau_{22}+u_{1}\tau_{12})\}. \end{array}$$

Here, $\hat{\beta }$ and $\hat{\lambda }$ are the MLEs of the parameters β and λ, and $\hat{H}(f)$ represents the MLE of H (f). The detailed derivation of these Bayesian estimates is shown in Appendix C.

#### 3.2.3. General Entropy Loss Function

Using Lindley’s approximation method, the Bayesian estimations of two parameters, β and λ, and the entropy under the GEL function can, respectively, be given by

$${\hat{\beta }}_{E}={\left\{{\hat{\beta }}^{-q}+0{.5[u}_{11}\tau_{11}{+u}_{1}\tau_{{}_{11}}^{2}z_{30}{+u}_{1}\tau_{21}\tau_{22}z_{03}{+3u}_{1}\tau_{11}\tau_{12}z_{21}+(\tau_{11}\tau_{22}{+2u}_{1}\tau_{21}^{2}{)u}_{1}z_{12}]+u_{1}\tau_{11}p_{1}+u_{1}\tau_{12}p_{2}\right\}}^{-\;1/q}$$

$${\hat{\lambda }}_{L}={\left\{{\hat{\lambda }}^{-\;q}+0{.5[u}_{22}\tau_{22}{+u}_{2}\tau_{11}\tau_{12}z_{30}{+u}_{2}\tau_{22}^{2}z_{03}+(\tau_{11}\tau_{22}+2\tau_{12}^{2}{)u}_{2}z_{21}+3u_{2}\tau_{22}\tau_{21}z_{21}]+u_{2}\tau_{21}p_{1}+u_{2}\tau_{22}p_{2}\right\}}^{-\;1/q}$$

(33) $$\begin{array}{l} {\hat{H}}_{E}(f)=\{{\left[\hat{H}(f)\right]}^{-q}+0{.5[(u}_{11}\tau_{11}{+2u}_{12}\tau_{12}+u_{22}\tau_{22}{)+z}_{30}{(u}_{1}\tau_{11}{+u}_{2}\tau_{12})\tau_{11}{+z}_{03}{(u}_{2}\tau_{22}{+u}_{1}\tau_{12})\tau_{22}\\ {+z}_{21}{(3u}_{1}\tau_{11}\tau_{12}{+u}_{2}(\tau_{11}\tau_{22}+2\tau_{12}^{2}{))+z}_{12}{(3u}_{2}\tau_{22}\tau_{21}{+u}_{1}(\tau_{11}\tau_{22}+2\tau_{21}^{2}))]\\ +p_{1}{(u}_{1}\tau_{11}+u_{2}\tau_{21})+p_{2}{(u}_{2}\tau_{22}+u_{1}\tau_{12}{)\}}^{-1/q}. \end{array}$$

Here, $\hat{\beta }$ and $\hat{\lambda }$ are the MLEs of the parameters β and λ, and $\hat{H}(f)$ represents the MLE of H (f). The detailed derivation of these Bayesian estimates is shown in Appendix D.

### 3.3. Bayesian Credible Interval

In the previous subsection, we used the Lindley’s approximation method to obtain the Bayesian point estimation of the parameters and entropy. However, this approximation method cannot determine the Bayesian CIs. Thus, the MCMC method is applied to obtain the Bayesian CI for the parameters and entropy. The MCMC method is a useful technique for estimating complex Bayesian models. The Gibbs sampling and Metropolis–Hastings algorithm are the two most frequently applied MCMC methods which are used in reliability analysis, statistical physics and machine learning, among other applications. Due to their practicality, they have gained some attention among researchers, and interesting results have been obtained. For example, Gilks and Wild [24] proposed adaptive rejection sampling to handle non-conjugacy in applications of Gibbs sampling. Koch [25] studied the Gibbs sampler by means of the sampling–importance resampling algorithm. Martino et al. [26] established a new approach, namely by recycling the Gibbs sampler to improve the efficiency without adding any extra computational cost. Panahi and Moradi [27] developed a hybrid strategy, combining the Metropolis–Hastings [28,29] algorithm with the Gibbs sampler to generate samples from the respective posterior, arising from the inverted, exponentiated Rayleigh distribution. In this paper, we adopt the method proposed in [27] to generate samples from the respective posterior arising from the GB distribution. From Equations (6) and (22), the joint posterior of the parameters β,λ can be written as

(34) $$\begin{array}{l} \pi (\beta ,\lambda |\vec{x})\propto \pi (\beta ,\lambda )L(\beta ,\lambda |\vec{x})\propto {\left[V(\lambda )\right]}^{m+a}\beta^{m+a-1}\exp[-\beta V(\lambda )]\prod_{i=1}^{m}\left[1-\exp(-\beta x_{i}^{\lambda })\right]\\ \times \frac{1}{{\left[V(\lambda )\right]}^{m+a}}\prod_{i=1}^{D}{\left(3-2\exp(-\beta x_{i}^{\lambda })\right)}^{R_{i}^{}}{\left(3-2\exp(-\beta x_{m}^{\lambda })\right)}^{R^{*}}\lambda^{m+c-1}\exp(-d\lambda )\prod_{i=1}^{m}x_{i}^{\lambda -1} \end{array}$$

Here, $V(\lambda )=(b+2\sum_{i=1}^{m}x_{i}^{\lambda }+2\sum_{i=1}^{D}R_{i}x_{i}^{\lambda }+2R^{*}x_{m}^{\lambda }).$ Therefore, we have

(35) $$\pi (\beta ,\lambda |\vec{x})\propto \pi_{1}(\beta |\lambda ,\vec{x})\pi_{2}(\lambda |\beta ,\vec{x}),$$

where

(36) $$\pi_{1}(\beta |\lambda ,\vec{x})\propto {\left[V(\lambda )\right]}^{m+a}\beta^{m+a-1}\exp[-\beta V(\lambda )]$$

(37) $$\begin{array}{ll} \pi_{2}(\lambda |\beta ,\vec{x}) & \propto \frac{\lambda^{m+c-1}}{{\left[V(\lambda )\right]}^{m+a}}\exp(-d\lambda )\exp[-\beta (2\sum_{i=1}^{m}x_{i}^{\lambda }+2\sum_{i=1}^{D}R_{i}x_{i}^{\lambda }+2R^{*}x_{m}^{\lambda })]\\ & \times \prod_{i=1}^{m}\left[1-\exp(-\beta x_{i}^{\lambda })\right]\prod_{i=1}^{D}{\left(3-2\exp(-\beta x_{i}^{\lambda })\right)}^{R_{i}^{}}{\left(3-2\exp(-\beta x_{m}^{\lambda })\right)}^{R^{*}}\prod_{i=1}^{m}x_{i}^{\lambda -1}. \end{array}$$

It is observed that the posterior density $\pi_{1}(\beta |\lambda ,\vec{x})$ of β, given λ, is the PDF of the Gamma distribution $Gamma(m+a,\;b+2\sum_{i=1}^{m}x_{i}^{\lambda }+2\sum_{i=1}^{D}R_{i}x_{i}^{\lambda }+2R^{*}x_{m}^{\lambda })$. However, the posterior density $\pi_{2}(\lambda |\beta ,\vec{x})$ of λ, given β, cannot be reduced analytically to a known distribution. Therefore, we use the Metropolis–Hastings method with normal proposal distribution to generate random numbers from Equation (37). We use the next algorithm (Algorithm 1), proposed in [27], to generate random numbers from Equation (34) and construct the Bayesian credible interval of λ, β and the entropy H (f).
**Algorithm 1** The MCMC method**Step 1:** Choose the initial value $\left(\beta^{\left(0\right)},\lambda^{\left(0\right)}\right)$.**Step 2:** At stage i and for the given m, n and ATII-PH censored data, generate $\beta^{\left(i\right)}$ from the following:$Gamma(m+a,\;b+2\sum_{i=1}^{m}x_{i}^{\lambda }+2\sum_{i=1}^{D}R_{i}x_{i}^{\lambda }+2R^{*}x_{m}^{\lambda }).$**Step 3:** Generate $\lambda^{\left(i\right)}$ from $\pi_{2}(\lambda^{\left(i-1\right)}|\beta^{\left(i\right)},\vec{x})$ using the following steps.**Step 3-1:** Generate $\lambda^{\prime }$ from $N(\lambda^{\left(i-1\right)},\mathrm{var}(\lambda ))$.**Step 3-2:** Generate the ω from the uniform distribution U(0, 1).**Step 3-3:** Set $\lambda^{\left(i\right)}=\{\begin{array}{c} \lambda^{\prime },\;\begin{array}{cc} & if\omega \leq r^{*} \end{array}\\ \lambda^{\left(i-1\right)},\begin{array}{ccc} & if\omega >r^{*} & \end{array} \end{array}$, where $r^{*}=\min\left\{1,\frac{\pi_{2}(\lambda^{\prime }|\beta^{\left(i\right)},\vec{x})}{\pi_{2}(\lambda^{\left(i-1\right)}|\beta^{\left(i\right)},\vec{x})}\right\}$.**Step 4:** Set i=i+1.**Step 5:** By repeating Steps 2–4 N times, we get $\left(\beta_{1},\lambda_{1}),(\beta_{2},\lambda_{2}),\ldots ,(\beta_{N},\lambda_{N}\right)$. Furthermore, we compute $H_{1},H_{2},\ldots ,H_{N}$, where $H_{i}=H(\beta_{i},\lambda_{i}),\;i=1,2,\ldots ,N$ and H(β,λ) is the Shannon entropy of the GB distribution.

Rearrange $(\beta_{1},\beta_{2},\ldots ,\beta_{N}),$ and $\left(H_{1},H_{2},\ldots ,H_{N}\right)$ into $(\beta_{\left(1\right)},\beta_{\left(2\right)},\ldots ,\beta_{\left(N\right)}),$ $\left(\lambda_{\left(1\right)},\lambda_{\left(2\right)},\ldots ,\lambda_{\left(N\right)}\right)$ and $\left(H_{\left(1\right)}H_{\left(2\right)},\ldots ,H_{\left(N\right)}\right)$, where $(\beta_{\left(1\right)}<\beta_{\left(2\right)}<\ldots <\beta_{\left(N\right)}),$ $\left(\lambda_{\left(1\right)}<\lambda_{\left(2\right)}<\ldots <\lambda_{\left(N\right)}\right)$ and $\left(H_{\left(1\right)}<H_{\left(2\right)}<\ldots <H_{\left(N\right)}\right)$.

Then, the 100(1-α)% Bayesian credible interval of the two parameters β,λ and the entropy are given by $\left(\beta_{\left(N\alpha /2\right)},\;\beta_{\left(N(1-\alpha /2)\right)}\right)$, $\left(\lambda_{\left(N\alpha /2\right)},\;\lambda_{\left(N(1-\alpha /2)\right)}\right)$ and $\left(H_{\left(N\alpha /2\right)},\;H_{\left(N(1-\alpha /2)\right)}\right)$.

## 4. Simulation Study

In this section, a Monte Carlo simulation study is carried out to observe the performance of different estimators of the entropy, in terms of the MSEs for different values of *n*, *m*, *T* and censoring schemes. In addition, the average 95% asymptotic confidence intervals (ACIs), Bayesian credible intervals (BCIs) of β,λ and the entropy, as well as the average interval length (IL), are computed, and the performances are also compared. We consider the following three different progressive censoring schemes (CSs):

- **CS I:**$R_{m}=n-m,\;R_{i}=0,i\neq m$;
- **CS II:**$R_{1}=n-m,R_{i}=0,i\neq 1$;
- **CS III:**$R_{m/2}=n-m,R_{i}=0,for\;i\neq \frac{m}{2},$ if m is even or $R_{(m+1)/2}=n-m,R_{i}=0,for\;i\neq \frac{m+1}{2},$ if m is odd.

Based on the following algorithm proposed by Balakrishnan and Sandhu [30] (Algorithm 2), we can generate an adaptive Type-II progressive hybrid censored sample from the GB distribution.
**Algorithm 2.** Generating a adaptive Type-II progressive hybrid censored sample from the GB distribution.**Step1:** Generate m independent observations $Z_{1},Z_{2},\ldots ,Z_{m}$, where $Z_{i}$ follows the uniform distribution U(0,1), i=1,2,…,m.**Step 2:** For the known censoring scheme $\left(R_{1},R_{2},\ldots ,R_{m}\right)$, let $\xi_{i}=Z_{i}{}^{1/(i+R_{m}+R_{m-1}+\ldots +R_{m-i+1})},i=1,2,\ldots ,m$.**Step 3:** By setting $U_{i}=1-\xi_{m}\xi_{m-1}\ldots \xi_{m-i+1}$, then $U_{1},U_{2},\ldots ,U_{m}$ is a Type-II progressive censored sample from the uniform distribution U(0,1).**Step 4:** Using the inverse transformation $X_{i:m:n}=F^{-1}(U_{i})$, i=1,2,…,m, we obtain a Type-II progressive censored sample from the GB distribution; that is, $X_{1:m:n},X_{2:m:n},\ldots ,X_{m:m:n}$, where $F^{-1}(\cdot )$ denotes the GB distribution’s inverse cumulative functional expression with the parameter (β,λ). The following theorem1 gives the uniqueness of the solution for the equation $X_{i:m:n}=F^{-1}(U_{i})$, i=1,2,…,m.**Step 5:** If there exists a real number J satisfying $X_{J:m:n}<T\leq X_{J+1:m:n}$, then we set index J and record $X_{1:m:n},X_{2:m:n},\ldots ,X_{J+1:m:n}$.**Step 6:** Generate the first m−J−1 order statistics $X_{J+2:m:n},X_{J+3:m:n},\ldots ,X_{m:m:n}$ from the truncated distribution $f(x;\beta ,\lambda )/[1-F(x_{J+1};\beta ,\lambda )]$ with a sample size $n-J-1-\sum_{i=1}^{J}R_{i}$.

**Theorem** **1.** *The equation $X_{i:m:n}=F^{-1}(U_{i})$ has a unique solution, i=1,2,…,m.*

**Proof.**  **See Appendix A.** □

In the simulation study, we took the values of the parameters of the GB distribution as *β* = 1, *λ* = 2. In this case, H(*f*) = 0.2448. The hyperparameter values of the prior distribution were taken as a=1,b=3,c=2,d=3. For the Linex loss function and general entropy loss function, we set h=−1.0,  1.0 and q=−1.0,  1.0, respectively. In the Newton iterative algorithm and MCMC sampling algorithm, we chose the initial values of β and λ as $\beta^{\left(0\right)}=0.9,\lambda^{\left(0\right)}=1.9;$ the value of ε was taken as $10^{-6}$. For different sample sizes n and different effective samples *m* and time *T*, we used 3000 simulated samples in each case. The average values and mean square errors (MSEs) of the MLEs and Bayesian estimations (BEs) for β,λ and the entropy were calculated. These results are reported in Table 1, Table 2, Table 3, Table 4, Table 5 and Table 6.

From Table 1, Table 2, Table 3, Table 4, Table 5 and Table 6, the following observations can be made:

1. For the fixed m and T values, the MSEs of the MLEs and Bayesian estimations of the two parameters and the entropy decreased when n increased. As such, we tended to get better estimation results with an increase in the test sample size;
2. For the fixed n and m values, when T increased, the MSEs of the MLEs and Bayesian estimations of the two parameters and the entropy did not show any specific trend. This could be due to the fact that the number of observed failures was preplanned, and no additional failures were observed when T increased;
3. In most cases, the MSEs of the Bayesian estimations under a squared error loss function were smaller than those of the MLEs. There was no significant difference in the MSEs between the Linex loss and general entropy loss functions;
4. For fixed values of n, m and T, Scheme II was smaller than Scheme I and Scheme III in terms of the MSE.

**Figure 1:**
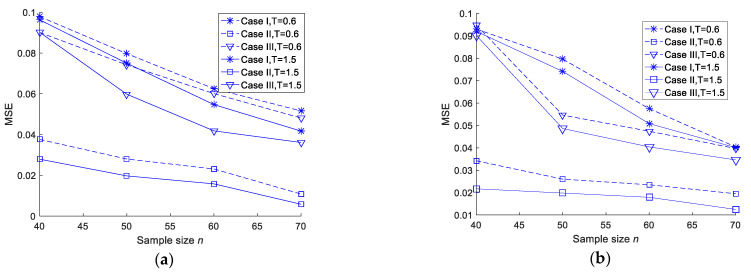

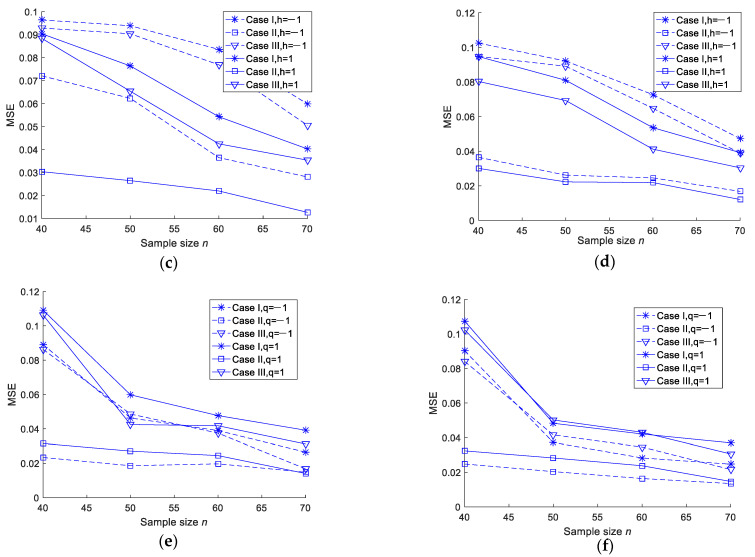
MSEs of different entropy estimations. (**a**) MSEs of MLEs of entropy in the case of T = 0.6 and T = 1.5. (**b**) MSEs of Bayesian estimations of entropy under a squared error loss function in the case of T = 0.6 and T = 1.5. (**c**) MSEs of Bayesian estimations of entropy under a Linex loss function in the case of T = 0.6. (**d**) MSEs of Bayesian estimations of entropy under a Linex loss function in the case of T = 1.5. (**e**) MSEs of Bayesian estimations of entropy under a general entropy loss function in the case of T = 0.6. (**f**) MSEs of Bayesian estimations of entropy under a general entropy loss function in the case of T = 1.5.

To further demonstrate the conclusions, the MSEs are plotted when the sample size increases under different censoring schemes. The trends are shown in Figure 1 (values come from Table 1, Table 2, Table 3, Table 4, Table 5 and Table 6).

Furthermore, the average 95% ACIs and BCIs of β,λ and the entropy, as well as the average lengths (ALs) and coverage probabilities of the confidence intervals, were computed. These results are displayed in Table A1, Table A2, Table A3 and Table A4 (See Appendix E).

From Table A1, Table A2, Table A3 and Table A4, the following can be observed:

1. The coverage probability of the approximate confidence intervals and Bayes credible intervals became bigger when n increased while m and T remain fixed;
2. For fixed values of n and m, when T increased, we did not observe any specific trend in the coverage probability of the approximate confidence intervals and Bayesian credible intervals;
3. For fixed values of n and T, the average length of the approximate confidence intervals and Bayesian credible intervals were narrowed down when n increased;
4. The average length of the Bayesian credible intervals was smaller than that of the asymptotic confidence intervals in most cases;
5. For fixed values of n and m, when T increased, we did not observe any specific trend in the average length of the confidence intervals;
6. For fixed values of n, m and T, Scheme II was smaller than Scheme I and Scheme III in terms of the average length of the credible interval;
7. For fixed values of n, m and T, the coverage probability of the approximate confidence intervals and Bayesian credible intervals were bigger than Scheme I and Scheme III.

## 5. Real Data Analysis

In this subsection, a real data set is considered to illustrate the use of the inference procedures discussed in this paper. This data set consisted of 30 successive values of March precipitation in Minneapolis–Saint Paul, which were reported by Hinkley [31]. The data set points are expressed in inches as follows: 0.32, 0.47, 0.52, 0.59, 0.77, 0.81, 0.81, 0.9, 0.96, 1.18, 1.20, 1.20, 1.31, 1.35, 1.43, 1.51, 1.62, 1.74, 1.87, 1.89, 1.95, 2.05, 2.10, 2.20, 2.48, 2.81, 3.0, 3.09, 3.37 and 4.75 in.

This data was used by Barreto-Souza and Cribari-Neto [32] for fitting the generalized exponential-Poisson (GEP) distribution and by Abd-Elrahman [20] for fitting the Bilal and GB distributions. In the complete sample case, the MLEs of *β* and *λ* were 0.4168 and 1.2486, respectively. In this case, we calculated the maximum likelihood estimate of the entropy as H(*f*) = 1.2786. For the above data set, Abd-Elrahman [20] pointed out that the negative of the log likelihood, Kolmogorov–Smirnov (K–S) test statistics and its corresponding p value related to these MLEs were 38.1763, 0.0532 and 1.0, respectively. Based on the value of p, it is clear that the GB distribution was found to fit the data very well. Using the above data set, we generated an adaptive Type-II progressive hybrid censoring scheme with an effective failure number m (m = 20).

When we took T = 4.0 and $R_{1}=R_{2}=\ldots =R_{5}=1,\;R_{6}=R_{7}=\ldots =R_{15}=0,\;$$R_{16}=R_{17}=\ldots =R_{20}=1,$ the obtained data in Case I were as follows:

Case I: 0.32, 0.52, 0.77, 0.81, 0.96, 1.18, 1.20, 1.31, 1.35, 1.43, 1.51, 1.62, 1.74, 1.87, 1.89, 1.95, 2.10, 2.48, 2.81 and 3.37.

When we took T = 2.0, $R_{1}=1,\;R_{2}=R_{3}=\ldots =R_{8}=0,$ $R_{9}=R_{10}=\ldots R_{15}=1,$ $R_{16}=R_{17}=\ldots =R_{19}=0$ and $R_{20}=2$, the obtained data in Case II were as follows:

Case II: 0.32, 0.47, 0.52, 0.59, 0.77, 0.81, 0.9, 0.96, 1.18, 1.20, 1.35, 1.43, 1.74, 1.87, 1.95, 2.10, 2.20, 2.48, 2.81 and 3.09.

Based on the above data, the maximum likelihood estimation and Bayesian estimation of the entropy and the two parameters could be calculated. For the Bayesian estimation, since we had no prior information about the unknown parameters, we considered the noninformative gamma priors of the unknown parameters as a = b = c = d = 0. For the Linex loss and general entropy functions, we set h=−1.0,   1.0 and q=−1.0,   1.0, respectively. The MLEs and Bayesian estimations of the entropy and the two parameters were calculated by using the Newton–Raphson iteration and Lindley’s approximation method. These results are tabulated in Table 7 and Table 8. In addition, the 95% asymptotic confidence intervals (ACIs) and Bayesian credible intervals (BCs) of the two parameters and the entropy were calculated using the Newton–Raphson iteration, delta method and MCMC method. These results are displayed in Table 9.

From Table 7, Table 8 and Table 9, we can observe that the MLEs and Bayesian estimations of the parameters and the entropy were close to the estimations in the complete sample case. In most cases, the length of the Bayesian credible intervals was smaller than that of the asymptotic confidence intervals.

## 6. Conclusions

In this paper, we considered the estimation of parameters and entropy for generalized Bilal distribution using adaptive Type-II progressive hybrid censored data. Using an iterative procedure and asymptotic normality theory, we developed the MLEs and approximate confidence intervals of the unknown parameters and the entropy. The Bayesian estimates were derived by Lindley’s approximation under the square, Linex and general entropy loss functions. Since Lindley’s method failed to construct the intervals, we utilized Gibbs sampling together with the Metropolis–Hastings sampling procedure to construct the Bayesian credence intervals of the unknown parameters and the entropy. A Monte Carlo simulation was provided to show all the estimation results. The results illustrate that the proposed methods performed well. The applicability of the considered model in a real situation was illustrated, based on the data of March precipitation in Minneapolis–Saint Paul. It was observed that the considered model could be utilized to analyze this real data appropriately.

## Figures and Tables

**Table 1:** The average maximum likelihood estimations (MLEs) and mean square errors (MSEs) of *β*, *λ* and the entropy (*β* = 1, *λ* = 2, H(*f*) = 0.2448).

(n, m)	SC	T = 0.6			T = 1.5		
		$\hat{\beta }$ MSE	$\hat{\lambda }$ MSE	$\hat{H}$ MSE	$\hat{\beta }$ MSE	$\hat{\lambda }$ MSE	$\hat{H}$ MSE
(40, 15)	I	1.1850 0.1224	2.2096 0.1428	0.1903 0.0979	1.1875 0.1213	2.2848 0.1521	0.1950 0.0963
	II	1.0727 0.0709	2.1448 0.1258	0.2015 0.0376	1.0619 0.0609	2.1541 0.1336	0.2017 0.0279
	III	1.1819 0.1217	2.2354 0.1413	0.1947 0.0910	1.1864 0.1208	2.2362 0.1514	0.1968 0.0902
(50, 15)	I	1.1326 0.1053	2.1803 0.1398	0.2086 0.0797	1.0905 0.0741	2.1931 0.1483	0.1997 0.0750
	II	1.0498 0.0390	2.1017 0.1243	0.2281 0.0280	1.0390 0.0374	2.1076 0.1263	0.2169 0.0197
	III	1.1184 0.1013	2.1817 0.1345	0.2035 0.0742	1.0740 0.0602	2.1284 0.1448	0.2013 0.0598
(60, 30)	I	1.1006 0.0889	2.1758 0.1374	0.2029 0.0625	1.0689 0.0683	2.1795 0.1368	0.2033 0.0547
	II	1.0451 0.0363	2.0847 0.1066	0.2260 0.0231	1.0476 0.0383	2.0877 0.1048	0.2170 0.0158
	III	1.0860 0.0653	2.1528 0.1368	0.2086 0.0601	1.0583 0.0592	2.1571 0.1335	0.2090 0.0418
(70, 30)	I	1.0641 0.0704	2.1296 0.1202	0.2163 0.0516	1.0581 0.0597	2.1197 0.1278	0.2134 0.0417
	II	1.0246 0.0265	2.0785 0.0849	0.2294 0.0198	1.0231 0.0317	2.0715 0.0946	0.2245 0.0148
	III	1.0517 0.0580	2.1483 0.1203	0.2199 0.0591	1.0468 0.0485	2.1132 0.1203	0.2195 0.0361

**Table 2:** The average Bayesian estimations and MSEs of *β*, *λ* and the entropy under the squared error loss functon (*β* = 1, *λ* = 2; *β* = 1, *λ* = 2, H(*f*) = 0.2448).

(n, m)	SC	T = 0.6			T = 1.5		
		$\hat{\beta }$ MSE	$\hat{\lambda }$ MSE	$\hat{H}$ MSE	$\hat{\beta }$ MSE	$\hat{\lambda }$ MSE	$\hat{H}$ MSE
(40, 15)	I	0.8625 0.0353	1.8735 0.1325	0.3357 0.0930	0.8687 0.0337	1.8761 0.1317	0.3301 0.0920
	II	0.9480 0.0235	1.9583 0.0954	0.2630 0.0342	0.9546 0.0255	1.9531 0.0938	0.2616 0.0217
	III	0.8795 0.0340	1.8041 0.1314	0.3264 0.0948	0.8837 0.0310	1.8996 0.1299	0.3034 0.0902
(50, 15)	I	0.9325 0.0297	1.8917 0.1185	0.3189 0.0796	0.8973 0.0289	1.8345 0.0975	0.2732 0.0741
	II	0.9645 0.0218	1.9907 0.0827	0.2580 0.0260	0.9694 0.0223	1.9763 0.0812	0.2303 0.0198
	III	0.9475 0.0253	1.9013 0.1072	0.3016 0.0546	0.9824 0.0234	1.9314 0.0972	0.2661 0.0486
(60, 30)	I	0.9274 0.0224	1.8445 0.1151	0.2357 0.0575	0.9457 0.0263	1.8781 0.0919	0.2674 0.0508
	II	0.9671 0.0202	1.9932 0.0728	0.2398 0.0235	0.9688 0.0207	2.0176 0.0741	0.2235 0.0179
	III	0.9185 0.0211	1.8525 0.1072	0.2301 0.0534	0.9316 0.0227	1.9427 0.0954	0.2652 0.0504
(70, 30)	I	0.9742 0.0198	1.9360 0.0775	0.2538 0.0404	0.9515 0.0213	1.9504 0.0892	0.2553 0.0401
	II	0.9895 0.0174	2.0413 0.0613	0.2506 0.0195	0.9804 0.0186	2.0378 0.0537	0.2260 0.0105
	III	0.9787 0.0182	1.9746 0.0761	0.2512 0.0397	0.9713 0.0194	1.9714 0.0683	0.2537 0.0346

**Table 3:** The average Bayesian estimations and MSEs of *β*, *λ* and the entropy under the Linex loss function (*β* = 1, *λ* = 2, T = 0.6, H(*f*) = 0.2448).

(n, m)	SC	h=−1			h=1		
		$\hat{\beta }$ MSE	$\hat{\lambda }$ MSE	$\hat{H}$ MSE	$\hat{\beta }$ MSE	$\hat{\lambda }$ MSE	$\hat{H}$ MSE
(40, 15)	I	0.8835 0.0355	1.8558 0.1261	0.3583 0.0964	0.8531 0.0366	1.8248 0.1343	0.2802 0.0904
	II	0.9740 0.0255	1.9161 0.0885	0.2587 0.0721	0.9308 0.0246	1.9092 0.1008	0.2469 0.0304
	III	0.9047 0.0308	1.8768 0.1249	0.3343 0.0929	0.8670 0.0335	1.8405 0.1889	0.2638 0.0884
(50, 15)	I	0.9047 0.0301	1.9415 0.1238	0.3158 0.0939	0.8704 0.0337	1.9175 0.1329	0.2736 0.0764
	II	0.9852 0.0218	2.0538 0.0789	0.2502 0.0623	0.9674 0.0213	1.9201 0.0912	0.2358 0.0265
	III	0.9105 0.0284	1.9771 0.0986	0.3046 0.0904	0.8924 0.0293	1.9203 0.1257	0.2604 0.0654
(60, 30)	I	0.9341 0.0223	1.9788 0.1127	0.2792 0.0836	0.9035 0.0238	1.9221 0.1308	0.2520 0.0543
	II	0.9834 0.0198	2.0465 0.0664	0.3743 0.0365	0.9609 0.0211	1.9447 0.0791	0.2118 0.0220
	III	0.9498 0.0204	1.9837 0.0973	0.3424 0.0829	0.9258 0.0207	1.9253 0.1227	0.2319 0.0425
(70, 30)	I	0.9561 0.0197	1.9889 0.0768	0.2546 0.0579	0.9378 0.0184	1.9543 0.0975	0.2407 0.0403
	II	0.9957 0.0174	2.0312 0.0572	0.2371 0.0281	0.9798 0.0159	2.0164 0.0614	0.2410 0.0187
	III	0.9687 0.0185	2.0024 0.0746	0.2265 0.0536	0.9451 0.0120	1.9623 0.0784	0.2409 0.0354

**Table 4:** The average Bayesian estimations and MSEs of *β*, *λ* and the entropy under the Linex loss function (*β* = 1, *λ* = 2, T = 1.5, H(*f*) = 0.2448).

(n, m)	SC	h=−1			h=1		
		$\hat{\beta }$ MSE	$\hat{\lambda }$ MSE	$\hat{H}$ MSE	$\hat{\beta }$ MSE	$\hat{\lambda }$ MSE	$\hat{H}$ MSE
(40, 15)	I	0.8896 0.0330	1.8328 0.1359	0.3492 0.1025	0.8510 0.0375	1.8127 0.1396	0.3381 0.0947
	II	0.9638 0.0248	1.9177 0.0863	0.2743 0.0365	0.9272 0.0265	1.9167 0.0982	0.2657 0.0301
	III	0.8922 0.0321	1.8691 0.1306	0.3424 0.0948	0.8631 0.0334	1.8430 0.1328	0.3343 0.0803
(50, 15)	I	0.9024 0.0234	1.8678 0.1094	0.3217 0.0921	0.8823 0.0315	1.8874 0.1173	0.3216 0.0810
	II	0.9713 0.0221	1.9401 0.0731	0.2601 0.0262	0.9418 0.0217	1.9824 0.0884	0.2632 0.0223
	III	0.9135 0.0231	1.8792 0.090	0.3383 0.0921	0.8975 0.0314	1.8845 0.1121	0.3210 0.0693
(60, 30)	I	0.9470 0.0219	1.8946 0.0951	0.3222 0.0727	0.9080 0.0234	1.9012 0.1075	0.3251 0.0536
	II	0.9795 0.0209	1.9452 0.0719	0.2518 0.0246	0.9548 0.0199	1.9616 0.0776	0.2513 0.0219
	III	0.9425 0.0213	1.8978 0.0906	0.3197 0.0648	0.9253 0.0213	1.9041 0.1069	0.3218 0.0412
(70, 30)	I	0.9583 0.0184	1.9562 0.0748	0.3165 0.0473	0.9491 0.0179	1.9493 0.0861	0.3314 0.0392
	II	0.9901 0.0163	2.0576 0.0652	0.2318 0.0168	0.9814 0.0153	2.0997 0.0608	0.2459 0.0161
	III	0.9711 0.0175	1.9230 0.0697	0.3027 0.0389	0.9502 0.0162	1.9894 0.0841	0.3267 0.0304

**Table 5:** The average Bayesian estimations and MSEs of *β*, *λ* and the entropy under the general entropy loss function (*β* = 1, *λ* = 2, T = 0.6, H(*f*) = 0.2448).

(n, m)	SC	q=−1			q=1		
		$\hat{\beta }$ MSE	$\hat{\lambda }$ MSE	$\hat{H}$ MSE	$\hat{\beta }$ MSE	$\hat{\lambda }$ MSE	$\hat{H}$ MSE
(40, 15)	I	0.8739 0.0341	1.8380 0.1348	0.3181 0.0891	0.8288 0.0437	1.8173 0.1381	0.3558 0.1091
	II	0.9546 0.0239	1.9184 0.0966	0.2832 0.0234	0.9169 0.0265	1.9081 0.1084	0.2628 0.0315
	III	0.8828 0.0324	1.8422 0.1306	0.3097 0.0863	0.8494 0.0389	1.8266 0.1361	0.3207 0.1063
(50, 15)	I	0.9013 0.0305	1.8948 0.1191	0.3017 0.0463	0.8972 0.0380	1.8728 0.1231	0.3423 0.0598
	II	0.9701 0.0214	1.9386 0.0803	0.2695 0.0186	0.9430 0.0236	1.9471 0.0962	0.2268 0.0271
	III	0.9251 0.0263	1.8984 0.1093	0.3023 0.0486	0.8613 0.0308	1.8498 0.1176	0.3287 0.0525
(60, 30)	I	0.9270 0.0232	1.9089 0.0824	0.2776 0.0390	0.8975 0.0276	1.8785 0.1127	0.3270 0.0477
	II	0.9610 0.0190	2.0351 0.0686	0.2318 0.0197	0.9481 0.0210	2.0453 0.0791	0.2391 0.0245
	III	0.9406 0.0210	1.9105 0.0874	0.2698 0.0375	0.9116 0.0231	1.8938 0.1109	0.3168 0.0418
(70, 30)	I	0.9501 0.0171	1.9492 0.0778	0.2536 0.0265	0.9213 0.0202	1.9308 0.0840	0.2924 0.0392
	II	0.9817 0.0158	2.0147 0.0436	0.2325 0.0148	0.9681 0.0151	2, 1489 0.0526	0.2410 0.0272
	III	0.9546 0.0174	1.9602 0.0738	0.2513 0.0168	0.9467 0.0173	1.9436 0.0724	0.2902 0.0312

**Table 6:** The average Bayesian estimations and MSEs of *β*, *λ* and the entropy under the general entropy loss function (*β* = 1, *λ* = 2, T = 1.5, H(*f*) = 0.2448).

(n, m)	SC	q=−1			q=1		
		$\hat{\beta }$ MSE	$\hat{\lambda }$ MSE	$\hat{H}$ MSE	$\hat{\beta }$ MSE	$\hat{\lambda }$ MSE	$\hat{H}$ MSE
(40, 15)	I	0.8770 0.0335	1.8569 0.1332	0.3564 0.0903	0.8224 0.0455	1.7924 0.1331	0.3598 0.1075
	II	0.9560 0.0218	1.9221 0.0914	0.2729 0.0198	0.9112 0.0257	1.9038 0.0913	0.2786 0.0294
	III	0.8836 0.0315	1.8297 0.1217	0.3519 0.0841	0.8453 0.0348	1.8374 0.1224	0.3547 0.1024
(50, 15)	I	0.8947 0.0298	1.8979 0.0981	0.3028 0.0372	0.8631 0.0362	1.8308 0.1134	0.3143 0.0483
	II	0.9685 0.0206	1.9793 0.0801	0.2610 0.0164	0.9377 0.0216	1.9467 0.0910	0.2656 0.0283
	III	0.8984 0.0278	1.9078 0.0931	0.3012 0.0416	0.8702 0.0302	1.8547 0.1086	0.3125 0.0502
(60, 30)	I	0.9244 0.0221	1.8446 0.0772	0.2731 0.0283	0.8930 0.0267	1.9208 0.1041	0.2812 0.0421
	II	0.9767 0.0188	2.0526 0.0614	0.2554 0.0164	0.9440 0.0202	2.0658 0.0718	0.2627 0.0238
	III	0.9387 0.0198	1.9541 0.0824	0.2709 0.0346	0.9125 0.0210	1.9435 0.0983	0.2801 0.0431
(70, 30)	I	0.9531 0.0167	1.9578 0.0738	0.2501 0.0247	0.9230 0.0188	1.9447 0.0814	0.2523 0.0370
	II	0.9814 0.0140	2.2263 0.0394	0.2309 0.0135	0.9675 0.0140	2.2680 0.0338	0.2352 0.0247
	III	0.9624 0.0163	1.9795 0.0745	0.2486 0.0216	0.9457 0.0164	1.9539 0.0718	0.2501 0.0306

**Table 7:** MLEs and Bayesian estimations of the parameters and the entropy.

MLEs	Case I	Case II	BEs (Squared Loss)	Case I	Case II
${\hat{\beta }}_{M}$	0.3289	0.3948	${\hat{\beta }}_{S}$	0.3428	0.4044
${\hat{\lambda }}_{M}$	1.0408	1.3373	${\hat{\lambda }}_{S}$	0.9974	1.2410
${\hat{H}}_{M}$	1.5890	1.3881	${\hat{H}}_{S}$	1.6230	1.4701

**Table 8:** Bayesian estimations of the parameters and the entropy under two loss functions.

BEs Linex Loss	h=−1		h=1		BEs Entropy Loss	q=−1		q=1	
	Case I	Case II	Case I	Case II		Case I	Case II	Case I	Case II
${\hat{\beta }}_{L}$	0.3406	0.4031	0.3330	0.3958	${\hat{\beta }}_{E}$	0.3369	0.4025	0.3273	0.3852
${\hat{\lambda }}_{L}$	1.2893	1.0217	1.2442	0.9898	${\hat{\lambda }}_{E}$	1.2618	1.0060	1.2173	0.9765
${\hat{H}}_{L}$	1.4714	1.6681	1.4385	1.6276	${\hat{H}}_{E}$	1.4608	1.6340	1.4370	1.6249

**Table 9:** The 95% asymptotic confidence intervals (ACIs) and Bayesian credible intervals (BCIs) with the corresponding interval lengths (ILs) of the two parameters and the entropy.

Parameter	ACIs IL		Parameter	BCIs IL	
	Case I	Case II		Case I	Case II
*β*	(0.2406, 0.5409) 0.3003	(0.1812, 0.4564) 0.2752	*β*	(0.2760, 0.5625) 0.2865	(0.2210, 0.4923) 0.2713
*λ*	(0.6899, 1.3918) 0.7019	(0.9884, 1.7863) 0.7979	*λ*	(0.7021, 1.3566) 0.6545	(0.8776, 1.6743) 0.7967
*H*	(1.2012, 1.9314) 0.7302	(1.0299, 1.7863) 0.7164	*H*	(1.2487, 1.9707) 0.7220	(1.1266, 1.8671) 0.7405

## Data Availability

Not available.

## Appendix A. Proof of Theorem 1

We set $y=\exp\;(-\beta x_{}^{\lambda }),$ then 0<y<1. The cumulative distribution function of GB distribution can be written as

$$F(x;\beta ,\lambda )=1-3y^{2}+2y^{3},0<y<1$$

By setting $u=1-3y^{2}+2y^{3},0<u<1$, then we get $3y^{2}-2y^{3}+u-1=0,$0<y<1.

Set $\rho (y)=3y^{2}-2y^{3}+u-1,$ take the first derivative of ρ(y) with respect to *y*, and we have $\frac{d\rho (y)}{dy}=6y-6y^{2}>0,$ as 0<y<1.

Notice that ρ(y) is a monotonically increasing function when 0<y<1. Thus, there is a unique solution to the equation $3y^{2}-2y^{3}+u-1=0$ when 0<y<1. As such, we have proven that the equation $X_{i:m:n}=F^{-1}(U_{i})$ has a unique solution (i=1,2,…,m).

## Appendix B. The Specific Steps of the Newton–Raphson Iteration Method

**Step 1:** Give the initial values of θ=(β,λ); that is, $\theta^{\left(0\right)}=(\beta^{\left(0\right)},\lambda^{\left(0\right)})$.

**Step 2:** In the *k*th iteration, calculate $\left(\frac{\partial l}{\partial \beta },\frac{\partial l}{\partial \lambda }\right)|{}_{\begin{array}{l} \beta =\beta^{\left(k\right)}\\ \lambda =\lambda^{\left(k\right)} \end{array}}$ and $I(\beta_{}^{\left(k\right)},\lambda^{\left(k\right)})$, where $I(\beta_{}^{\left(k\right)},\lambda^{\left(k\right)})=\left[\begin{array}{cc} I_{11} & I_{12}\\ I_{21} & I_{22} \end{array}\right]|{}_{\begin{array}{l} \beta =\beta^{\left(k\right)}\\ \lambda =\lambda^{\left(k\right)} \end{array}}$ is the observed information matrix of the parameters β and λ, and $I_{ij},i=1,2,3$ are given by Equations (10)–(13).

**Step 3:** Update ${\left(\beta ,\lambda \right)}^{T}$ with

${\left(\beta^{\left(k+1\right)},\lambda^{\left(k+1\right)}\right)}^{T}={\left(\beta^{\left(k\right)},\lambda^{\left(k\right)}\right)}^{T}+I^{-1}(\beta^{\left(k\right)},\lambda^{\left(k\right)})\times {\left(\frac{\partial l}{\partial \beta },\frac{\partial l}{\partial \lambda }\right)}^{T}|{}_{\begin{array}{l} \beta =\beta^{\left(k\right)}\\ \lambda =\lambda^{\left(k\right)} \end{array}}.$

Here, ${\left(\beta ,\lambda \right)}^{T}$ is the transpose of vector (β,λ), and $I^{-1}(\beta^{\left(k\right)},\lambda^{\left(k\right)})$ represents the inverse of the matrix $I(\beta^{\left(k\right)},\lambda^{\left(k\right)})$.

**Step 4:** Setting k=k+1, the MLEs of the parameters (denoted by $\hat{\beta }$ and $\hat{\lambda }$) can be obtained by repeating Steps 2 and 3 until $\left|{\left(\beta^{\left(k+1\right)},\lambda^{\left(k+1\right)}\right)}^{T}-{\left(\beta^{\left(k\right)},\lambda^{\left(k\right)}\right)}^{T}\right|<\varepsilon$, where ε is a threshold value that is fixed in advance.

## Appendix C. The Detailed Derivation of Bayesian Estimates of two Parameters (β,λ) and the Entropy under the LL Function

In this case, we take U(β,λ)=exp(−hβ), and then

$$u_{1}=-h\exp\;(-h\beta ),u_{11}=h^{2}\exp\;(-h\beta ),u_{12}=u_{21}=u_{22}=u_{2}=0.$$

Using Equation (26), the Bayesian estimation of parameter β is given by

$${\hat{\beta }}_{L}=-\frac{1}{h}\ln\{\exp(-h\hat{\beta })+0{.5[u}_{11}\tau_{11}{+u}_{1}\tau_{{}_{11}}^{2}z_{30}{+u}_{1}\tau_{21}\tau_{22}z_{03}{+3u}_{1}\tau_{11}\tau_{12}z_{21}+(\tau_{11}\tau_{22}{+2u}_{1}\tau_{21}^{2}{)u}_{1}z_{12}]+u_{1}\tau_{11}p_{1}+u_{1}\tau_{12}p_{2}\}$$

Similarly, the Bayesian estimation of parameter λ is obtained by

$${\hat{\lambda }}_{L}=-\frac{1}{h}\ln\{\exp(-h\hat{\lambda })+0{.5[u}_{22}\tau_{22}{+u}_{2}\tau_{11}\tau_{12}z_{30}{+u}_{2}\tau_{22}^{2}z_{03}+(\tau_{11}\tau_{22}+2\tau_{12}^{2}{)u}_{2}z_{21}+3u_{2}\tau_{22}\tau_{21}z_{21}]+u_{2}\tau_{12}p_{1}+u_{2}\tau_{22}p_{2}\}$$

For the Bayesian estimation of the entropy, we have

$$\begin{array}{l} U(\beta ,\lambda )=\exp[-hH(f)],u_{1}=\frac{h}{\beta \lambda }\exp[-hH(f)],\\ u_{2}=-h[-\frac{1}{\lambda }+\frac{1}{\lambda^{2}}(\ln\beta -\ln(9/8)+\gamma )]\exp[-hH(f)],\\ u_{11}=h[\frac{-1}{\beta^{2}\lambda }+\frac{h}{\beta^{2}\lambda^{2}}]\exp[-hH(f)], \end{array}$$

$$u_{22}=\{-h\;[\frac{1}{\lambda^{2}}-\frac{2}{\lambda^{3}}(\ln\beta -\ln(9/8)+\gamma )]+h^{2}{\left[-\frac{1}{\lambda }+\frac{1}{\lambda^{2}}(\ln\beta -\ln(9/8)+\gamma )\right]}^{2}\}\exp[-hH(f)],$$

$$u_{12}=u_{21}==h[\frac{h-1}{\beta \lambda^{2}}-h\frac{1}{\beta \lambda^{3}}(\ln\beta -\ln(9/8)+\gamma )]\exp[-hH(f)].$$

The Bayesian estimation of the entropy under the LL function is given by

$$\begin{array}{l} {\hat{H}}_{L}(f)=-\;\frac{1}{h}\ln\{\exp[-h\hat{H}(f)]+0{.5[u}_{11}\tau_{11}{+2u}_{12}\tau_{12}+u_{22}\tau_{22}{+z}_{30}{(u}_{1}\tau_{11}{+u}_{2}\tau_{12})\tau_{11}{+z}_{03}{(u}_{2}\tau_{22}{+u}_{1}\tau_{21})\tau_{22}\\ {+z}_{21}{(3u}_{1}\tau_{11}\tau_{12}{+u}_{2}(\tau_{11}\tau_{22}+2\tau_{12}^{2}{))+z}_{12}{(3u}_{2}\tau_{22}\tau_{21}{+u}_{1}(\tau_{11}\tau_{22}+2\tau_{21}^{2}))]\\ +p_{1}{(u}_{1}\tau_{11}+u_{2}\tau_{21})+p_{2}{(u}_{2}\tau_{22}+u_{1}\tau_{12})\} \end{array}$$

## Appendix D. The Derivation of Bayesian Estimates of two Parameters (β,λ) and the Entropy under the GEL Function

In this case, we take $U(\beta ,\lambda )=\beta^{-q}$ and then $u_{1}=-q\beta^{-q-1},\;u_{11}=q(q+1)\beta^{-q-2},$ and $u_{12}=u_{21}=u_{22}=u_{2}=0.$

Using Equation (26), the Bayesian estimation of parameter β is given by

$${\hat{\beta }}_{E}={\left\{{\hat{\beta }}^{-q}+0{.5[u}_{11}\tau_{11}{+u}_{1}\tau_{{}_{11}}^{2}z_{30}{+u}_{1}\tau_{21}\tau_{22}z_{03}{+3u}_{1}\tau_{11}\tau_{12}z_{21}+(\tau_{11}\tau_{22}{+2u}_{1}\tau_{21}^{2}{)u}_{1}z_{12}]+u_{1}\tau_{11}p_{1}+u_{1}\tau_{12}p_{2}\right\}}^{-\;1/q}$$

Similarly, the Bayesian estimation of parameter λ is obtained by

$${\hat{\lambda }}_{L}={\left\{{\hat{\lambda }}^{-\;q}+0{.5[u}_{22}\tau_{22}{+u}_{2}\tau_{11}\tau_{12}z_{30}{+u}_{2}\tau_{22}^{2}z_{03}+(\tau_{11}\tau_{22}+2\tau_{12}^{2}{)u}_{2}z_{21}+3u_{2}\tau_{22}\tau_{21}z_{21}]+u_{2}\tau_{21}p_{1}+u_{2}\tau_{22}p_{2}\right\}}^{-\;1/q}$$

For the Bayesian estimation of the entropy under the general EL function, we take $U(\beta ,\lambda )={\left[H(f)\right]}^{-q},$ and then

$$u_{1}=\frac{q}{\beta \lambda }{\left[H(f)\right]}^{-q-1},\;u_{2}=[\frac{q}{\lambda }-\frac{q}{\lambda^{2}}(\ln\beta -\ln(9/8)+\gamma )]{\left[H(f)\right]}^{-q-1},$$

$$\begin{array}{l} u_{2}=[\frac{q}{\lambda }-\frac{q}{\lambda^{2}}(\ln\beta -\ln(9/8)+\gamma )]{\left[H(f)\right]}^{-q-1},u_{11}=\frac{q(q+1)}{\beta^{2}\lambda^{2}}{\left[H(f)\right]}^{-q-2}-\frac{q}{\beta^{2}\lambda }{\left[H(f)\right]}^{-q-1},\\ u_{22}=[\frac{-q}{\lambda^{2}}+\frac{2q}{\lambda^{3}}(\ln\beta -\ln(9/8)+\gamma )]{\left[H(f)\right]}^{-q-1}+q(q+1){\left[\frac{1}{\lambda }-\frac{1}{\lambda^{2}}(\ln\beta -\ln(9/8)+\gamma )\right]}^{2}{\left[H(f)\right]}^{-q-2},\\ u_{12}=u_{21}=q(q+1)[\frac{1}{\beta \lambda^{2}}-\frac{1}{\beta \lambda^{3}}(\ln\beta -\ln(9/8)+\gamma )]{\left[H(f)\right]}^{-q-2}-\frac{q}{\beta \lambda^{2}}{\left[H(f)\right]}^{-q-1}. \end{array}$$

Using Equation (26), the approximate Bayesian estimation of the entropy is given by

$$\begin{array}{l} {\hat{H}}_{E}(f)=\{{\left[\hat{H}(f)\right]}^{-q}+0{.5[(u}_{11}\tau_{11}{+2u}_{12}\tau_{12}+u_{22}\tau_{22}{)+z}_{30}{(u}_{1}\tau_{11}{+u}_{2}\tau_{12})\tau_{11}{+z}_{03}{(u}_{2}\tau_{22}{+u}_{1}\tau_{12})\tau_{22}\\ {+z}_{21}{(3u}_{1}\tau_{11}\tau_{12}{+u}_{2}(\tau_{11}\tau_{22}+2\tau_{12}^{2}{))+z}_{12}{(3u}_{2}\tau_{22}\tau_{21}{+u}_{1}(\tau_{11}\tau_{22}+2\tau_{21}^{2}))]\\ +p_{1}{(u}_{1}\tau_{11}+u_{2}\tau_{21})+p_{2}{(u}_{2}\tau_{22}+u_{1}\tau_{12}{)\}}^{-1/q}. \end{array}$$

## Appendix E

**Table A1:** The average 95% approximate confidence intervals and average lengths and coverage probabilities of *β*, *λ* and the entropy (*β* = 1, *λ* = 2, H(*f*) = 0.2448, T = 0.6).

(n, m)	SC	***β*** AL	CP	***λ*** AL	CP	H AL	CP
(40, 15)	I	(0.6598, 1.5736) 0.9138	0.9042	(1.2220, 3.1773) 1.9573	0.9216	(0.0293, 1.1866) 1.1573	0.9184
	II	(0.6711, 1.4742) 0.8031	0.9253	(1.4238, 2.8658) 1.4420	0.9361	(0.0393, 0.7733) 0.7340	0.929
	III	(0.6343, 1.5347) 0.9004	0.9130	(1.2645, 3.1064) 1.9319	0.9281	(0.0254, 1.1244) 1.0990	0.9174
(50, 15)	I	(0.6421, 1.5458) 0.9037	0.9162	(1.2837, 3.0913) 1.8076	0.9314	(0.0203, 1.0469) 1.0266	0.9216
	II	(0.7102, 1.3884) 0.6782	0.9394	(1.4416, 2.7246) 1.2830	0.9406	(0.0438, 0.6924) 0.6486	0.9392
	III	(0.6914, 1.5147) 0.8233	0.9253	(1.3021, 2.9705) 1.6684	0.9370	(0.0264, 1.0759) 1.0495	0.9261
(60, 30)	I	(0.6377, 1.5335) 0.8958	0.9374	(1.3388, 3.0191) 1.6803	0.9487	(0.0151, 0.9112) 0.8959	0.9393
	II	(0.7093, 1.3769) 0.6676	0.9516	(1.4807, 2.6886) 1.2069	0.9542	(0.0536, 0.6667) 0.6131	0.9461
	III	(0.6934, 1.4786) 0.7852	0.9405	(1.3955, 2.9630) 1.5675	0.9506	(0.0325, 0.8630) 0.8305	0.9428
(70, 30)	I	(0.7329, 1.4293) 0.6964	0.9472	(1.4068, 2.8432) 1.34364	0.9534	(0.0298, 0.7943) 0.7645	0.9446
	II	(0.7247, 1.2859) 0.5602	0.9651	(1.5369, 2.5891) 1.0522	0.9680	(0.0614, 0.5498) 0.4884	0.9632
	III	(0.7392, 1.3486) 0.6154	0.9514	(1.4476, 2.7845) 1.3361	0.9573	(0.0498, 0.7185) 0.6687	0.9521

**Table A2:** The average 95% approximate confidence intervals and average lengths and coverage probabilities of *β*, *λ* and the entropy (*β* = 1, *λ* = 2, H(*f*) = 0.2448, T = 1.5).

(n, m)	SC	***β*** AL	CP	***λ*** AL	CP	H AL	CP
(40, 15)	I	(0.5234, 1.8717) 1.3483	0.9231	(1.2469, 3.2287) 1.9818	0.9274	(0.0284, 1.1887) 1.1603	0.9267
	II	(0.6662, 1.4576) 0.7914	0.9372	(1.4322, 2.8760) 1.4438	0.9405	(0.0436, 0.7887) 0.7451	0.9393
	III	(0.5619, 1.8110) 1.2491	0.9252	(1.2679, 3.2045) 1.9364	0.9364	(0.0212, 1.1173) 1.0961	0.9340
(50, 15)	I	(0.5601, 1.6810) 1.1209	0.9230	(1.3076, 3.0214) 1.7136	0.9363	(0.0245, 0.9304) 0.9059	0.9347
	II	(0.7124, 1.3705) 0.6581	0.9418	(1.4548, 2.7213) 1.2665	0.9462	(0.0458, 0.6740) 0.6282	0.9515
	III	(0.6103, 1.5868) 0.9765	0.9336	(1.3320, 2.9769) 1.6449	0.9372	(0.0259, 0.8461) 0.8202	0.9347
(60, 30)	I	(0.6659, 1.5135) 0.8476	0.9418	(1.3454, 3.0335) 1.6881	0.9521	(0.0206, 1.0400) 1.0194	0.9464
	II	(0.7051, 1.3680) 0.6619	0.9592	(1.4812, 2.6942) 1.2130	0.9574	(0.0456, 0.6604) 0.6148	0.9531
	III	(0.6913, 1.4513) 0.7600	0.9431	(1.3775, 2.8768) 1.4983	0.9520	(0.0237, 0.9934) 0.9697	0.9506
(70, 30)	I	(0.7381, 1.3951) 0.6570	0.9492	(1.4501, 2.7820) 1.3319	0.9582	(0.0321, 0.7553) 0.7232	0.9523
	II	(0.7573, 1.2850) 0.5277	0.9704	(1.5514, 2.5845) 1.0331	0.9726	(0.0647, 0.5680) 0.5033	0.9741
	III	(0.7554, 1.3492) 0.5938	0.9546	(1.4967, 2.7071) 1.2104	0.9615	(0.0410, 0.7147) 0.6737	0.9591

**Table A3:** The average 95% Bayesian credible intervals and average lengths and coverage probabilities of *β*, *λ* and the entropy (*β* = 1, *λ* = 2 H(*f*) = 0.2448, T = 0.6).

(n, m)	SC	***β*** AL	CP	***λ*** AL	CP	H AL	CP
(40, 15)	I	(0.5521, 1.2841) 0.7320	0.9194	(1.0215, 2.4593) 1.4378	0.9241	(0.0213, 1.1750) 1.1537	0.9263
	II	(0.6378, 1.3228) 0.6850	0.9433	(1.2854, 2.5238) 1.2384	0.9472	(0.0395, 0.7752) 0.7357	0.9380
	III	(0.5670, 1.2953) 0.7283	0.9253	(1.0579, 2.4762) 1.4183	0.9294	(0.0224, 1.1192) 1.0968	0.9308
(50, 15)	I	(0.5924, 1.2871) 0.6947	0.9312	(1.1731, 2.5054) 1.3323	0.9397	(0.0298, 0.9231) 0.8933	0.9386
	II	(0.6897, 1.2921) 0.6024	0.9491	(1.3580, 2.4935) 1.1355	0.9465	(0.0548, 0.6751) 0.6203	0.9507
	III	(0.6067, 1.2854) 0.6787	0.9342	(1.2051, 2.4718) 1.2667	0.9354	(0.0278, 0.8553) 0.8275	0.9326
(60, 30)	I	(0.6450, 1.2925) 0.6475	0.9481	(1.1389, 2.4565) 1.3176	0.9536	(0.0397, 1.0509) 1.0112	0.9394
	II	(0.6870, 1.2905) 0.6035	0.9614	(1.3883, 2.4740) 1.0857	0.9656	(0.0578, 0.6717) 0.6139	0.9562
	III	(0.6565, 1.2812) 0.6247	0.9532	(1.1919, 2.4423) 1.2504	0.9561	(0.0319, 0.8408) 0.8029	0.9528
(70, 30)	I	(0.7062, 1.2494) 0.5432	0.9512	(1.3068, 2.4374) 1.1306	0.9563	(0.0324, 0.7516) 0.7192	0.9536
	II	(0.7451, 1.2449) 0.4998	0.9711	(1.4821, 2.4494) 0.9673	0.9744	(0.0701, 0.5672) 0.4971	0.9783
	III	(0.7162, 1.2359) 0.5197	0.9583	(1.3597, 2.4443) 1.0846	0.9604	(0.0440, 0.7067) 0.6627	0.9578

**Table A4:** The average 95% Bayesian credible intervals and average lengths and coverage probabilities of *β*, *λ* and the entropy (*β* = 1, *λ* = 2 H(*f*) = = 0.2448, T = 1.5).

(n, m)	SC	***β*** AL	CP	***λ*** AL	CP	H AL	CP
(40, 15)	I	(0.5554, 1.2954) 0.7400	0.9218	(1.0243, 2.4612) 1.4369	0.9354	(0.0251, 1.1801) 1.1550	0.9258
	II	(0.6417, 1.3339) 0.6922	0.9439	(1.2824, 2.5169) 1.2345	0.9485	(0.0372, 0.7728) 0.7356	0.9394
	III	(0.5696, 1.3033) 0.7337	0.9275	(1.0556, 2.4672) 1.4116	0.9318	(0.0241, 1.1200) 1.0959	0.9337
(50, 15)	I	(0.5954, 1.2947) 0.6993	0.9417	(1.1722, 2.4804) 1.3002	0.9420	(0.0224, 1.0231) 1.0007	0.9418
	II	(0.68902, 1.2954) 0.6062	0.9506	(1.3599, 2.5034) 1.1435	0.9525	(0.0479, 0.6710) 0.6239	0.9526
	III	(0.6045, 1.2801) 0.6756	0.9359	(1.2337, 2.5094) 1.2757	0.9364	(0.0324, 1.0047) 0.9723	0.9371
(60, 30)	I	(0.6418, 1.2835) 0.6417	0.9494	(1.1349, 2.4455) 1.3106	0.9548	(0.0250, 0.9212) 0.8960	0.9417
	II	(0.6896, 1.2970) 0.6074	0.9628	(1.3987, 2.4911) 1.0924	0.9662	(0.0479, 0.6608) 0.6129	0.9573
	III	(0.6600, 1.2856) 0.6256	0.9556	(1.1549, 2.4283) 1.2734	0.9571	(0.0217, 0.8359) 0.8142	0.9538
(70, 30)	I	(0.7061, 1.2472) 0.5411	0.9526	(1.3179, 2.4521) 1.1342	0.9571	(0.0363, 0.7509) 0.7146	0.9548
	II	(0.7451, 1.2413) 0.4962	0.9725	(1.4663, 2.4268) 0.9605	0.9757	(0.0778, 0.5701) 0.4923	0.9793
	III	(0.7154, 1.2267) 0.5113	0.9594	(1.3542, 2.4118) 1.0576	0.9624	(0.0604, 0.7108) 0.6504	0.9585
